# Assessment of *Cucurbita* spp. Peel Extracts as Potential Sources of Active Substances for Skin Care and Dermatology

**DOI:** 10.3390/molecules27217618

**Published:** 2022-11-06

**Authors:** Katarzyna Gaweł-Bęben, Karolina Czech, Marcelina Strzępek-Gomółka, Marcin Czop, Monika Szczepanik, Anna Lichtarska, Wirginia Kukula-Koch

**Affiliations:** 1 Department of Cosmetology, University of Information Technology and Management, Sucharskiego 2, 35-225 Rzeszów, Poland; 2 Department of Clinical Genetics, Medical University of Lublin, Radziwiłłowska 11, 20-080 Lublin, Poland; 3 Department of Pharmacognosy with Medicinal Plants Garden, Medical University of Lublin, Chodźki 1, 20-093 Lublin, Poland

**Keywords:** *Cucurbita* spp., peel extract, natural resources, antioxidant, tyrosinase, sun protection factor, in vitro cytotoxicity

## Abstract

By-products of cultivated plants are one of the major environmental concerns worldwide. Due to the high concentration of bioactive chemicals, such waste may be considered hazardous due to the interference with the plant growth, deterioration of the drinking water quality or toxic effects on sensitive marine organisms. Moreover, plant-derived by-products, with proper handling, may represent a low-cost source of bioactive compounds potentially important for pharmaceutical and cosmetics industries. The aim of the study was to evaluate the phytochemical composition, antioxidant activity, the influence of tyrosinase activity, in vitro sun protecting factor and cytotoxicity of 15 extracts from peels of five cultivars of *Cucurbita maxima* and *C. moschata*. The extracts were prepared using “green solvents” (water, 50% propylene glycol, and 20% ethanol) and ultrasound-assisted extraction. The performed analysis showed that the peel extracts from various cultivars differ significantly in respect to the phytochemical content and activity. The type of solvent also had a significant impact on the extract’s composition and bioactivity. Aqueous peel extracts contained the highest amounts of flavonoids, showed the greatest antioxidant potential and the most significant in vitro SPF values. In vitro studies showed that the analyzed peel extracts are not cytotoxic for human keratinocytes up to the concentration of 1000 µg/mL and thus might be considered as non-irritant for the skin. The study confirms the potential application of peel extracts from *Cucurbita* spp. cultivars in cosmetic products.

## 1. Introduction

The growing demand for extracts and substances of plant origin in the food, pharmaceutical and cosmetics industries contributes to the generation of an increasing amount of biodegradable waste, which may constitute from 10% to even 60% of the plant material. These wastes are mainly parts of plants that are not consumed, not used for industrial purposes or arise in the processing of vegetables and fruits, such as stems, leaves, seeds, hulls, roots or pomace obtained during juicing or pressing oils [1,2]. By-products from agricultural and food processing industries have become a serious ecological issue, due to the possibility of leading to environmental pollution and generating significant costs related to its storage and disposal. Throwing away of plant-derived biomass also raises questions about the rational exploitation of natural resources. As proven by several examples, agricultural and food processing by-products contain considerable quantities of valuable bioactive compounds and therefore can be useful for technological and pharmaceutical purposes [3,4,5]. Agricultural by-products are also considered as a rich source of active compounds for cosmetic applications. The utilization of this source of raw material is especially interesting for the cosmetics market, due to the growing consumer interests in “zero-waste” and sustainable cosmetics [6,7].

Pumpkin (*Cucurbita* L.) is an economically important species with a high production rate. It belongs to the *Cucurbitaceae* family, which comprises about 130 species growing in the wild and cultivated all over the world. There are about 20 species belonging to the *Cucurbita* genus, including the most commonly cultivated: *Cucurbita maxima* Duchesne, *C. pepo* L., *C. moschata* Duchesne ex Poir, *C. fificolia* Bouché, and *C. argyrosperma* C. Huber. Yellow to dark orange colors of *Cucurbita* sp. fruits result from the high content of carotenoids, including carotene, lutein or zeaxanthin. These two pigments absorb UV radiation and blue light, as well as scavenge free radicals and reactive oxygen species (ROS) [8]. The most commonly used part of the pumpkin is the pulp, used for the production of various food products and as a source of natural pigment in the form of powder added to confectionery, bakery, pasta, and dairy products. Pumpkin pulp and seeds are also sources of various phytochemicals with documented health-promoting properties, including antioxidant, antimicrobial, and anticancer activities [9]. Extracts, juices, and powders from the whole fruits of *Cucurbita* spp. are also well known active ingredients in cosmetics with skin conditioning, hair conditioning, humectant, and skin protecting functions [10].

The chemical composition of pumpkin pulp is considerably diversified and it depends both on the species and variety. Kulczyński and Gramza-Michałowska, compared the content of carotenoids, polyphenols, flavonoids, tocopherols, minerals, vitamins C and B1, and folates in the pulp of 15 cultivars of two pumpkin species, *C. pepo* L. and *C*. *moschata* [11] and the content of carotenoids, polyphenols, tocopherols, minerals, and vitamins in fruits of 11 *C. maxima* Duchesne cultivars [12]. Kostecka-Gugała et al. compared the chemical composition of the pulp of fruits of 18 cultivars of four species: *Cucurbita maxima*, *C. pepo*, *C. moschata* and *C. ficifolia* [13]. All mentioned studies showed significant differences in the total concentration of bioactive compounds, as well as the content of particular phytochemicals between pumpkin cultivars.

Pumpkin peel is a less studied part of the fruit but recent scientific data indicate that peels from various cultivars are also rich in biologically active phytochemicals, such as carotenoids, polyphenolic compounds, and amino acids [14,15,16]. Pumpkin peel extracts were shown to possess specific biological activities. Shaygan et al. showed that the treatment of burn wounds in rats with cream containing hydroalcoholic extracts from the peel of *C. moschata,* improved the parameters associated with efficient wound repair, including a better regeneration of the epidermic layer, a higher density of dermis collagen fibers, and lower presence of inflammatory cells, indicating its regenerative potential [15]. Accelerated wound healing and a reduced expression of tissue oxidative stress biomarkers by *C. moschata* peel extracts in rat skin wound models was also reported by Bahramsoltani and colleagues [16]. These data indicate a possible application on *Cucurbita* spp. peel extracts in dermatology and skin care products. To the best of our knowledge, a detailed analysis of the phytochemical content of the peel from different *Cucurbita* species and varieties, as well as a comparison of their biological activities, has not been described in the scientific literature, to date.

In order to meet consumer expectations, the preparation of plant extracts for the purpose of the cosmetics industry must follow green extraction procedures, characterized by a low energy consumption and a high recovery of active compounds. Ultrasound-assisted extraction (UAE) and microwave-assisted extraction (MAE) are good examples of such methods, which allow for the efficient extraction of active substances from the plant material [17,18,19]. Application of non-toxic solvents, which can be easily evaporated or allowed to be used directly in cosmetic products, is another important factor to be considered. These solvents include water, ethanol, or mixtures of water with propylene glycol (PG) or glycerine [20]. Sharma and colleagues compared the efficacy of the carotenoid extraction from the peel of *Cucurbita maxima* var. Gold Nugget and Amoro F1, using three extraction technologies considered as green—UAE, MEA, and conventional solvent extraction. UAE was shown as the most efficient method [14]. Therefore, the aim of the following study was to evaluate the composition and selected biological activities of aqueous (A), hydroglycolic (HG), and hydroethanolic (E) extracts from peels of five cultivars of *Cucurbita maxima* and *C. moschata*, prepared using UAE, in respect of their application as active ingredients for the cosmetics industry.

## 2. Results and Discussion

### 2.1. Active Compounds of Cosmetic Significance Present in Cucurbita *sp.* Peel Extracts

Aqueous (W), hydroglycolic (HG) and hydroethanolic (E) extracts were prepared using ultrasound assisted extraction (UAE) and compared for their content of total phenolic compounds, flavonoids, and proteins (Table 1). UAE was found to be more efficient than the microwave assisted extraction and conventional solvent extraction in obtaining pumpkin peel extracts enriched in phenolic components, as well as its significant antioxidant activity [14].

The content of total phenolic compounds in the analyzed extracts that were calculated for the herein proposed extraction methodology, varied from 17.599 ± 0.124 to 4.623 ± 0.082 mg GAE/g dw. The E extract from *C. maxima* ‘Halloween’ and the W extract from *C. maxima* ‘Hokkaido’ contained the most significant amounts of these compounds. In respect to the flavonoids, the highest content of these compounds was found in the W extracts from all peels. The flavonoid content in the W extracts varied from 2.598 ± 0.127 (*C. moschata* ‘Muscat’) to 7.108 ± 0.120 (*C. maxima* ‘Hokkaido’) mg QE/g dw. The W extracts were also shown to contain the most significant amounts of proteins—the highest protein content was detected in *C. moschata* ‘Muscat’ (76.143 ± 4.261 mg BSA/g dw) and the ‘Nelson’ (68.695 ± 7.790 mg BSA/g dw) peel extracts. Previous studies by Achilonu et al. demonstrated that the content of protein in pumpkin peel is higher than in the flesh. In the mentioned study, the content of protein in the peel of *C. maxima* was 16.54 ± 2.69 g/kg raw weight and in *C. moschata* 11.30 ± 0.99 g/kg raw weight [21].

The content of particular phytochemicals in the W, HG and E extracts from the analyzed *C. maxima* and *C. moschata* peel extracts was also analyzed using a HPLC-ESI-QTOF-MS/MS instrument to deliver a list of tentatively identified components (Table 2, Table 3, Table 4, Table 5 and Table 6, Tables S1 and S2 in the Supplementary File).

The above tables and Tables S1 and S2 from the Supplementary File show the composition of the studied W, HG and E extracts obtained from the five selected varieties of pumpkins, the fragmentation spectra of the tentatively identified compounds and the fingerprints of all analyzed extracts. The spectrometric data were collected in a data-dependent method, in two operation modes (negative and positive ionization settings) to enrich the compositional data. The high-resolution mass spectra of all samples were however found to be quite poor. Among the metabolites identified in the applied conditions, fatty acids, phenolic acids, flavonoids, and amino acids were distinguished. The richest qualitative profile was described for the ethanolic extracts. The water extracts were the main sources of amino acids, whereas the water-propylene glycol extracts had an intermediate composition between the other two solvents. These findings are in line with the previous publications, e.g., with the study of Koch and colleagues, who noticed that the addition of ethanol to water induced the content of phenolic acids [26], and with the previous studies of Kaczorova and colleagues [27], who selected ethanol extracts as the richest source of polyphenols from two *Achillea* spp.

To the best of our knowledge, comparative studies of the polyphenolic and flavonoid content in the extracts from peels of various pumpkin species and cultivars, have not been published, to date. However, significant variations in the content of individual phenolic acids and flavonols were observed in the extracts from whole fruits of pumpkin varieties belonging to the species *C. maxima*, *C. pepo,* and *C. moschata*. The most abundant phenolic acids found in fruit extracts were caffeic, ferulic, and gallic acids. Protocatechuic, 4-hydroxybenzoic, vanillic, p-coumaric, and sinapic acids were also detected in several pumpkin variety extracts. *C. pepo* varieties contained significantly higher amounts of caffeic acid and lower amounts of protocatechuic, p-coumaric, and synaptic acids than the *C. moschata* varieties [11,12,28]. Rutin was the flavonol found in all analyzed cultivars (the content of 51.92 ± 0.03 to 5.09 ± 0.01 mg/100 g dm). Kaempferol, quercetin, isoquercetin, astragalin, and myricetin were detected only in some of the varieties. No significant differences in the content of the listed flavonols was found between the *C. pepo* and *C. moschata* cultivars, except for quercetin, that was most abundant in *C. pepo* (3.29 ± 3.43 vs. 1.07 ± 1.74 mg/100 g dm) [12].

In our analysis, all pumpkin peel extracts were rich in various amino acids, including Tyr, Phe, Val, Pro Trp, Cys, and Arg, indicating their potential cosmetic application. Amino acids are major components of the skin’s natural moisturizing factor (NMF) and play an important role in regulating skin hydration and skin pH to keep skin healthy. Amino acids have been widely used in cosmetic skin care products, mostly for a skin hydration benefit. Application of an amino acid complex containing taurine, arginine, and glycine showed regenerative potential in an in vitro scratch assay using HaCaT cells and also using a 3D reconstructed human tissue model. The complex reduced skin irritation by decreasing the levels of IL-1α and also reduced skin redness and skin irritation in human studies [29].

Furthermore, the investigated samples are good sources of fatty acids—both saturated, such as palmitic acid, but also unsaturated ones, such as palmitoleic acid, oleic acid, and linoleic acid, that were found in the analyzed extracts in high quantities. As previously described, fatty acids induce a positive effect towards the skin cells by their protective and anti-inflammatory actions [30]. In the former studies, palmitoleic acid, the omega-7 monounsaturated acid that was proven to regulate the cytokine and type IV collagen levels [31], linoleic acid, the omega-6 polyunsaturated fatty acid that was found to be an efficient emollient and thickening agent [32], and palmitic acid, one of the most prevalent saturated oils of vegetable origin, were described as ingredients in hair- and skin-care products for their moisturizing and texturizing properties [33].

Based on the above information, the presence of fatty acids in the studied samples is beneficial and may support the cosmetic properties of the pumpkin peel extracts.

### 2.2. Antioxidant Properties of the Cucurbita *sp.* Peel Extracts

The antioxidant potential of *Cucurbita* spp. peel extracts was compared, using ABTS and DPPH scavenging assays (Table 7). In both assays, the highest activity was measured for the *C. moschata* ‘Butternut’ W extracts (4.524 ± 0.231 and 3.333 ± 0.004 µg TE/g dw the for ABTS and DPPH assays, respectively). The lowest antioxidant activity was detected for the HG extracts from *C. moschata* ‘Muscat’ (2.470 ± 0.041 µg TE/g dw for the ABTS assay) and *C. moschata* ‘Butternut” (0.947 ± 0.026 µg TE/g dw for the DPPH assay). For the most analyzed pumpkin varieties, the W and E peel extracts showed higher antioxidant activities than the HG extracts in both the ABTS and DPPH scavenging assays.

The antioxidant potential of pumpkin peel extracts has not been compared, to date. More data is available for total pumpkin fruit extracts. Kulczyński et al. determined the antioxidant activity of aqueous and aqueous-methanolic extracts from lyophilized flesh of 19 C. pepo and *C. moschata* cultivars, using the DPPH and ABTS scavenging methods, FRAP (ferric reducing antioxidant power), chelating activity, and ORAC. No statistically significant differences (*p* > 0.05) were found between the two pumpkin species (*C. pepo* vs. *C. moschata*), in respect to all of the used methods. However, significant differences were detected between extracts from particular pumpkin cultivars. Particularly a high antioxidant activity was found for ‘Delicata’, ‘Baby Boo’, and ‘Cream of the Crop’ cultivars of *C. pepo*. In addition, the mentioned study provided evidence of a higher antioxidant activity of the methanol-water extracts measured in the DPPH, FRAP, and chelating activity tests. A stronger antiradical activity in the ABTS cation radical test, as well as a higher total polyphenols content were observed for the aqueous extracts [11,12]. In another study, the antioxidant activity of 18 cultivars, belonging to *C. maxima*, *C. moschata*, *C. pepo,* and *C. ficifolia* species were compared, based on the FRAP, CUPRAC (cupric ion reducing antioxidant capacity), and DPPH assays. Among the analyzed samples, the ‘Hokkaido’ *C. maxima* extract exhibited the highest antioxidant and antiradical capacities [13].

The antioxidant properties of pumpkin extracts cannot be directly related to the content of total phenolics or flavonoids, shown in Table 1. Several other compounds with an antioxidant potential, which were not included in this study are more likely to present in the analyzed extracts: carotenoids, carbohydrates, and vitamins C and E [11,12]. Therefore, it is very difficult to estimate the total antioxidant activity of the pumpkin peel extract only, based on the content of the particular group of compounds.

### 2.3. The Influence of Cucurbita *spp.* Peel Extracts on the Tyrosinase Activity

Tyrosinase is the rate-limiting enzyme triggering the synthesis of melanin. Therefore it is a major target of most skin lightening cosmetics and medicines, used to treat hyperpigmentation disorders [34]. Moreover, the compounds activating tyrosinase might be applied in the topical treatment of vitiligo and other hypopigmentation disorders [35]. The influence of the pumpkin peel extracts on the tyrosinase activity was investigated, using commercially available mushroom tyrosinase, and murine tyrosinase contained in B16F10 melanoma cell lysate. A mushroom tyrosinase activity assay is the most widely used method of investigating the skin lightening potential of plant extracts. However, due to the structural and functional differences between the mushroom and mammalian tyrosinases, experimental models utilizing mammalian enzyme are of a greater physiological value [36].

**Figure 1:**
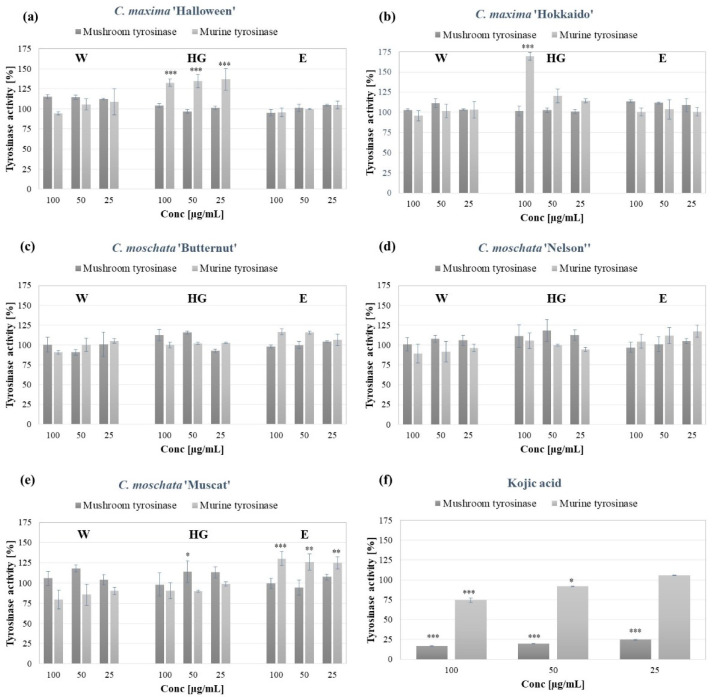
Mushroom and murine tyrosinase activities in the presence of the W (aqueous), HG (hydroglycolic) and E (ethanolic) extracts from *C. maxima* “Halloween” (**a**) and ‘Hokkaido” (**b**), *C. moschata* ‘Butternut’ (**c**), ‘Nelson’ (**d**) and ‘Muscat’ (**e**) cultivar peel extracts, in comparison with kojic acid (**f**); graphs show the mean tyrosinase activity ± SD, *n* = 3, * *p* < 0.05, ** *p* < 0.01, *** *p* < 0.001.

As shown in Figure 1, most of the studied extracts (except for the *C. moschata* ‘Muscat’ at 50 µg/mL), neither decrease nor increase the activity of mushroom tyrosinase. No inhibitory activity of the extracts was detected with regards to murine tyrosinase. However, the HG extracts from the peels of *C. maxima* ‘Halloween’ and ‘Hokkaido’, as well as the E extract from *C. moschata* ‘Muscat’, significantly increased the activity of the murine enzyme by 25–75%. The increased activity of tyrosinase, in the presence of pumpkin peel extracts might be partially explained by the content of the natural tyrosinase substrate, L-tyrosine, in most of the extracts. However, with respect to the HG extract from *C. maxima* ‘Halloween’ that did not contain L-tyrosine, there are most likely other compounds responsible for the observed tyrosinase activation.

The influence of pumpkin extracts on tyrosinase activity has not been extensively studied, to date. Recently, Chao et al. investigated the intracellular murine tyrosinase inhibitory activity of the polyphenols extract obtained from *C. maxima* flesh, which obtained a 31.44–37.12% inhibitory rate. The polyphenolic extract at concentrations >200 µg/mL was also shown to inhibit the intracellular activity of tyrosinase and the synthesis of melanin in B16F10 murine melanoma cells, however it also significantly reduced the viability of the cells at concentrations > 100 µg/mL. [37] Kikuchi and colleagues isolated the multiflorane-type triterpene from *C. maxima* seeds, which showed inhibitory effects on the α-MSH-induced melanogenesis in the B16 melanoma cell line [38] The skin-lightening potential of *Cucurbita* spp. was also investigated, with respect to the red pumpkin (*C. maxima*) seed extract. Using in vitro models, Endo and colleagues demonstrated that the extract suppressed the melanosome transfer to keratinocytes stimulated by ROS and generated following UVB exposure through the activation of the Nrf2 signaling [39].

### 2.4. In Vitro Cytotoxicity

**Figure 2:**
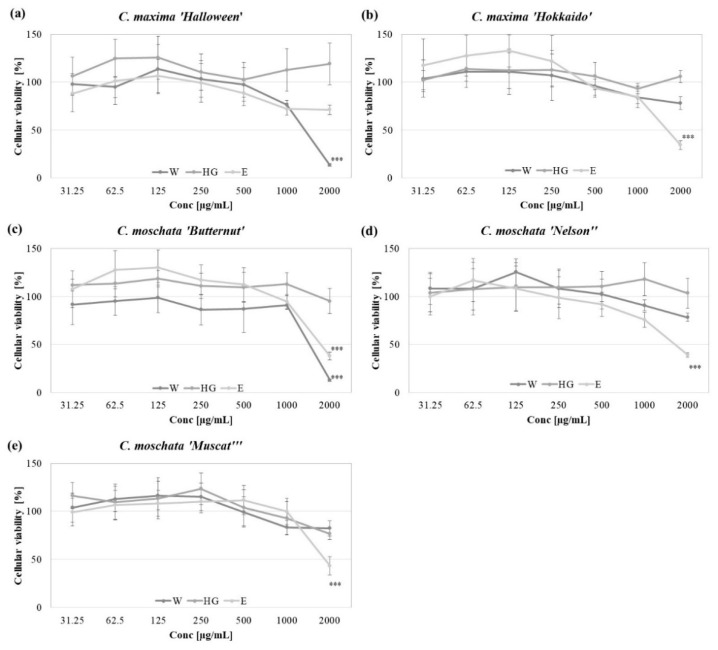
In vitro cytotoxicity of W (aqueous), HG (hydroglycolic) and E (ethanolic) extracts from the *C. maxima* “Halloween” (**a**), and ‘Hokkaido” (**b**), *C. moschata* ‘Butternut’ (**c**), ‘Nelson’ (**d**) and ‘Muscat’ (**e**) cultivar peel extracts on human keratinocytes HaCaT, following 48 h culture; graphs show the mean viability of the cells ± SD in comparison with the appropriate solvent controls; *** *p* < 0.001.

The potential application of pumpkin peel extracts, as active ingredients in cosmetic formulations, requires toxicological studies, in order to confirm their safety and lack of irritation potential. The cytotoxic effect of the analyzed peel extracts was investigated in vitro, using immortalized human keratinocytes HaCaT [40]. As shown in Figure 2, none of the analyzed extracts significantly decreased the viability of the HaCaT keratinocytes, following 48 h exposure, up to the concentration of 1000 µg/mL. The HG extracts did not significantly influence the viability of the keratinocytes, even at the highest tested concentration (2000 µg/mL), whereas the E extracts from the peels of the tested *C. maxima* and *C. moschata* varieties at 2000 µg/mL decreased the number of viable cells by about 40%. The cytotoxic effect was also observed with respect to the W extracts obtained from the peels of *C. maxima* ‘Halloween’ and *C. moschata* ‘Butternut’ at 2000 µg/mL.

The cytotoxic effect of the pumpkin peel extracts has been investigated, to date, only using human prostate [41] and liver [42] cancer cell lines. The extracts from whole fruits of the *C. pepo* ‘Lungo Fiorentino” (zucchini) has been previously shown to be cytotoxic for the HaCaT keratinocytes at a concentration of >200 µg/mL [43].

### 2.5. In Vitro Sun Protection Factor (SPF) of Pumpkin Peel Extracts

In addition to a few beneficial health effects (synthesis of vitamin D3, mood improvement), ultraviolet radiation (UVR) causes many detrimental skin effects, including sunburn, immune suppression, photoaging, DNA damage, and increases the risk of melanoma and non-melanoma skin cancer occurrences. Most of the harmful effects of UVR are mediated by the oxidative stress and induced expression of pro-inflammatory genes. UVR also causes immunosuppression by reducing the number and activities of the epidermal Langerhans cells [44,45]. Due to the harmful effects of UVR, sunscreens are commonly incorporated into the formulations of cosmetic products. The efficacy of a sunscreen is usually expressed by the sun protection factor (SPF), defined as the UV energy required for producing a minimal erythema dose (MED) on protected skin, divided by the UV energy required for producing MED on unprotected skin [46]. As the use of synthetic sunscreens raises some concerns, regarding their safety, natural products are currently extensively explored as safe alternative photoprotective ingredients [47]. One of the methods used to screen plant extracts for their photoprotective potential, is the determination of in vitro SPF, based on the Mansur equation and the absorbance measurements [48]. As shown in Table 8, among the analyzed samples, the W extracts showed the highest in vitro SPF values, varying from 7.59 ± 0.83 (*C. moschata* ‘Butternut’) to 2.56 ± 0.04 (*C. moschata* ‘Muscat’) at 1 mg/mL. For the *C. maxima* ‘Halloween’ peel extract, the highest in vitro SPF was measured for the E extract (7.30 ± 0.35). *Cucurbita* spp. preparations have been previously used as photoprotective ingredients in topical applications. In the studies, mostly the pumpkin seed oil was shown as an effective UV protecting ingredient, due to its sunscreen and antioxidant properties [49].

### 2.6. Corelation of the Pumpkin Peel Extract’s Activity with the Content of the Compounds

The bioactivity of peel extracts from pumpkin varieties has been correlated with the phytochemical content (Table 9). Among the W extracts, a positive correlation was shown between the content of flavonoids and the activity of murine tyrosinase (r = 0.553), a negative correlation between the total content of polyphenols and DPPH (r = −0.808), a negative correlation between the content of proteins and DPPH (r = −0.721) and murine tyrosinase activity (r = −0.550), a positive correlation between the protein content and cytotoxic activity (r = 0.516).

Among the HG extracts, negative correlations were found between the content of the flavonoids and DPPH (r = −0.649) and the activity of the mushroom tyrosinase (r = −0.797), positive correlations between the amount of polyphenols and ABTS (r = 0.651), DPPH (r = 0.694), SPF (r = 0.932) and the murine tyrosinase activity (r = 0.790), a positive correlation between the amount of proteins and DPPH (r = 0.713) and SPF (r = 0.797).

Among the E extracts, negative correlations were found between the content of the flavonoids and DPPH (r = −0.689) and the activity of the murine tyrosinase (−0.761), a positive correlation between the amount of polyphenols and SPF (0.830), a positive correlation between the amount of proteins and DPPH (r = 0.850) and the murine tyrosinase activity (r = 0.618).

### 2.7. PCA and Clustering Analysis

**Figure 3:**
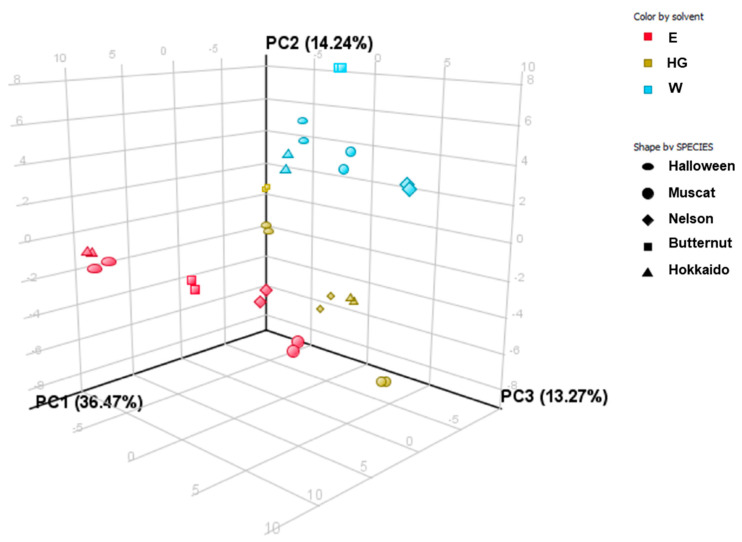
Scores of the first three principal components of the PCA explaining 63.98% of the variability in the obtained dataset. Explanation of the abbreviations: aqueous extracts (W), hydroglycolic extracts (HG), hydroethanolic extracts (E).

Following the principal component analysis (PCA), three components (PC) were distinguished, which together explain 63.98% of the variability of the original data. The first component explains 36.47% of the variation, the second component explains 14.24% of the variation, and the third component explains 13.27% of the variation (Figure 3).

When analyzing the results obtained in the PCA analysis, it can be concluded that the W extracts of all five varieties are distinctly different from the other extracts. The most similar extract is the HG extract of *C. moschata* ‘Butternut’. Additionally, two E extracts (*C. maxima* ‘Halloween’ and ‘Hokkaido’) definitely differ from all analyzed types of extracts. Moreover, the HG extract of *C. moschata* ‘Muscat’ also differs significantly from the other extracts.

**Figure 4:**
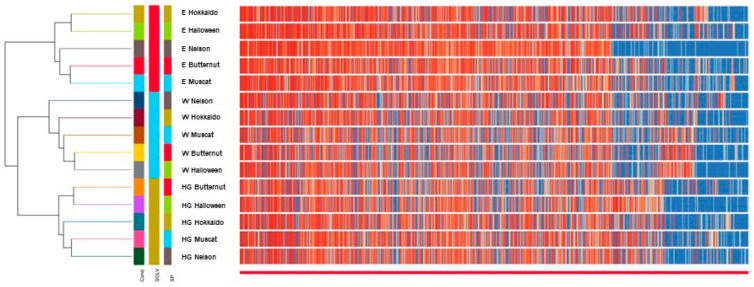
Dendrogram showing the results of the cluster analysis. Explanation of the abbreviations: aqueous extracts (W), hydroglycolic extracts (HG), hydroethanolic extracts (E).

The performed cluster analysis mostly confirms the conclusions of the PCA analysis, which proves that the type of solvent has a large impact on the qualitative composition of the obtained extracts (Figure 4).

## 3. Materials and Methods

### 3.1. Chemicals and Reagents

Propylene glycol (PG, >99.8% purity), Folin–Ciocalteu reagent and K_2_S_2_O_8_ were obtained from Chempur (Piekary Śląskie, Poland). Ethanol (EtOH, >99.8% purity) was obtained from Honeywell. Trolox, quercetin (>95% purity), gallic acid (>97.5% purity), 2,2-diphenyl-1-picrylhydrazyl (DPPH), 2,2’-azino-bis(3-ethylbenzothiazoline-6-sulfonic acid) (ABTS), tyrosinase from *Agaricus bisporus*, 3,4-dihydroxy-l-phenylalanine (L-DOPA), kojic acid (≥98.5% purity), TiO_2_, Dulbecco’s phosphate buffered saline (DPBS), Dulbecco’s modified Eagle’s medium (DMEM), bovine serum albumin (BSA), and Neutral Red Solution in DPBS (3.3 g/L) were purchased from Sigma Aldrich (Merck, Darmstadt, Germany). Fetal bovine serum (FBS) was obtained from Pan-Biotech (Aidenbach, Germany). The solvents used for the compositional study of the extracts by HPLC-MS (water, acetonitrile and formic acid) and were purchased from Merck (Darmstadt, Germany).

### 3.2. Plant Material and Extraction Procedure

Fruits of the *Cucurbita maxima* ‘Halloween’ and ‘Hokkaido’ and the *Cucurbita moschata* ‘Butternut’, ‘Nelson’ and ‘Muskat” cultivars were purchased from the local supplier (Podkarpackie, Poland). The fruits were peeled, using a standard vegetable peeler, in order to obtain the plant material corresponding to the commonly generated vegetable waste. The peels were dried using a laboratory dryer with air circulation (POL-EKO-APARATURA, Wodzisław Śląski, Poland) in the temperature not exceeding 40 °C. 1 g of the dried, ground plant material was mixed with 100 mL of water (W extracts), 20% (*v/v*) PG in a water mixture (HG extracts) or 70% (*v/v*) C_2_H_5_OH (E extracts) and was subjected to ultrasound-assisted extraction for 3 h, using an ultrasonic bath (Sonic-3, Polsonic, Warsaw, Poland). The extracts were filtered through the Whatman filter paper, 2 µm pre-filter and a 0.45 µm nylon syringe filter and evaporated using the Concentrator Plus system (Eppendorf, Warszawa, Poland) and stored at 4 °C, until analysis. The voucher specimen of the dried peel from each cultivar is retained in the Department of Cosmetology, University of Information Technology and Management in Rzeszów, Poland by the authors of the manuscript.

### 3.3. Analysis of the Total Phenolics and Flavonoids

The content of the total phenolic compounds was determined, as described by Fukumoto and Mazza [50] with modifications. Briefly, 150 μL of the extracts (1 mg/mL) was mixed with 750 μL of Folin–Ciocalteu reagent (1:10 *v/v*, in distilled water) and incubated for 5 min at room temperature in the darkness. The samples were mixed with 600 μL 7.5% (*m/v*) Na_2_CO_3_ and incubated for 1 h at room temperature in the darkness. The absorbance was measured at λ = 740 nm, using a DR 600 Spectrophotometer (Hach Lange, Wrocław, Poland). The calibration curves (y = 0.0113x − 0.0453, R^2^ = 0.9950 for HG 4:1; y = 0.014x − 0.0539, R^2^ = 0.9908 for 70% EtOH; y = 0.6412x − 0.6458, R^2^ = 0.9998 for the distilled water) were prepared using 0–100 mg/mL gallic acid in water, water-PG 4:1 and 70% ethanol. The content of the total phenolics was calculated as the gallic acid equivalents (GAEs) in mg per g of dried extract weight (dw).

The content of the flavonoids was measured, according to the method described by Matejić et al. [51]. First, 150 μL of the extract (10 mg/mL) was mixed with 650 μL of the reaction mixture (61.5 mL 80% C_2_H_5_OH + 1.5 mL 10% Al(NO_3_)_3_·9H2O + 1.5 mL 1 M CH_3_COOK). Following 40 min incubation at room temperature in the darkness, the absorbance of the samples was measured at λ = 415 nm. The calibration curves (y = 0.0087x + 0.0285, R^2^ = 0.9947 for HG 4:1; y = 0.0143x + 0.0109, R^2^ = 0.9991 for 70% EtOH; y = 0.0079x + 0.0161, R^2^ = 0.9927 for distilled water) were prepared using 0–100 mg/mL quercetin in water, water-PG 4:1 and 70% EtOH. The content of the flavonoids is expressed as quercetin equivalents (QuE) per gram of dw.

### 3.4. The HPLC-ESI-Q-TOF-MS Analysis of the Extracts

The HPLC-ESI-QTOF-MS/MS platform from Agilent Technologies (Santa Clara, CA, USA) was used to analyze the composition of all of the extracts. HPLC chromatograph (1200 series) with a Zorbax Eclipse Plus RP-18 chromatographic column (150 mm × 2.1 mm; dp = 3.5 µm) were applied in the study. The chromatograph was composed of a degasser (G1322A), a binary pump (G1312C), an autosampler (G1329B), a photodiode array detector (G1315D) and a mass spectrometer—QTOF with electrospray ionization (G6530B). Agilent MassHunter workstation software (version B.10.00) was used to acquire the MS spectra and process the data in a data-dependent method.

The following HPLC conditions were applied: the thermostat temperature was set at 25 °C, the UV detection wavelengths at 254, 280, 320, and 365 nm, the UV detector range as 190–600 nm, and the injection volume at 10 µL. The separation on the chromatographic column was achieved at a flow rate of 0.2 mL/min in a 50 min gradient elution program. The mobile phases consisted of eluent A (0.1% formic acid in water, *v/v*) and eluent B (acetonitrile solution with 0.1% formic acid added). The gradient elution was as follows: 0–2 min 1% B, 5 min 10% B, 8 min 30% B, 28 min 45% B, 30–34 min 95% B, and 35 min 1%B. The mass spectrometer measurements were carried out under the following conditions: gas temperature and shield gas temperature were 350 and 325 °C, respectively; the gas flow values: 12 L/min; capillary voltage of 3500 V; fragmentor voltage of 150 V; collision energies CID of 10 and 20 V; skimmer voltage current of 65 V; nebulizer pressure was set at 35 psig. The collected spectra were scanned in the *m/z* 40–1000 Da range in both the negative and positive ionization modes. Two of the most intense signals seen in the TIC spectrum were automatically fragmented to obtain the MS/MS spectra. Following the collection of two spectra for a given *m/z* value, the selected signals were excluded for 0.2 min from further fragmentation. The identification of the extracts’ components was performed, based on the high-resolution *m/z* measurements, the retention time, the fragmentation patterns, the scientific literature, and open mass databases (e.g., Metlin).

### 3.5. DPPH and ABTS Radical Scavenging Assays

The antiradical activity of the extracts was determined by the DPPH and ABTS radical scavenging assays [51]. For the DPPH scavenging assay, 100 μL of diluted extracts (0.0005–1 mg/mL) was mixed with 100 μL of methanolic DPPH solution (25 mM; A540~1); 100 μL of the solvent (water, 70% EtOH or HG 4:1) mixed with 100 μL DPPH, was used as a control sample. Then, after 20 min incubation at room temperature in the darkness, the absorbance of the samples was measured at λ = 540 nm, using a FilterMax F5 microplate reader (Molecular Devices, San Jose, CA, USA). The obtained values of the measurements were corrected by the absorbance values of the samples without DPPH. The Trolox calibration curve (y = 16.016x + 2.4046, R^2^ = 0.9900) was used to calculate the micrograms of Trolox equivalents per gram of dried extract weight (TE/g dw).

For the ABTS radical scavenging assay, the ABTS working solution was prepared by dissolving 7 mM ABTS in 2.45 mM K_2_S_2_O_8_ (A405~1); 15 μL of the extracts diluted in the appropriate solvent in the concentration range from 0.0005 to 1 mg/mL, was mixed with 135 μL ABTS working solution. Fifteen μL of the solvent mixed with 135 μL ABTS, served as a control sample. Following 15 min incubation at room temperature in the darkness, the absorbance of the samples was measured at λ = 405 nm, using a microplate reader (FilterMax F5, Molecular Devices, San Jose, CA, USA). The obtained values were corrected by the absorbance value of the sample without ABTS. The Trolox calibration curve (y = 25.3548x + 3.2926, R^2^ = 0.9900) was used to calculate the micrograms of Trolox equivalents per gram of dried extract weight (TE/g dw).

### 3.6. Tyrosinase Activity Assay

The influence of the *Cucurbita* sp. peel extracts on the activity of tyrosinase was compared using commercially available mushroom tyrosinase and murine tyrosinase, contained in the lysate of B16F10 murine melanoma cells (ATCC CRL-6475; LGC Standards, Łomianki, Poland), prepared as previously described [52]. The assay was performed, based on the method by Uchida and colleagues [53]. Briefly, 120 μL phosphate buffer (100 mM, pH = 6.8) was mixed with 20 μL of the diluted extracts (1, 0.5 and 0.25 mg/mL) and 20 μL of the mushroom tyrosinase (500 U/mL) and pre-incubated at room temperature for 10 min. Following the addition of 40 μL L-DOPA (4 mM) the samples were incubated for another 20 min at RT. The activity of the murine tyrosinase was assessed by mixing the volume of the cell lysate containing 20 µg protein with 20 µL of the diluted extract (1, 0.5 and 0.25 mg/mL), 40 µL 4 mM L-DOPA and 100 mM phosphate buffer pH 6.8 (up to 200 µL). The reaction was carried out for a 4 h incubation at 37 °C. The control sample (100% tyrosinase activity) for both assays contained the appropriate volume of the solvent instead of the extract. In both assays, the dopachrome formation was measured spectrophotometrically at λ = 450 nm, using a FilterMax F5 microplate reader (FilterMax F5 Molecular Devices, USA). The obtained values were corrected by the absorbance value of the extracts without the mushroom or murine tyrosinase and L-DOPA. Each sample was analyzed in three independent repetitions. Kojic acid was used as a known tyrosinase inhibitor control.

### 3.7. In Vitro Cytotoxicity

The cytotoxicity of pumpkin peels extracts was examined, using the neutral red uptake test [54]. HaCaT human keratinocytes cells (CLS Cell Lines Service GmbH, Eppelheim, Germany) were maintained in DMEM supplemented with 10% (*v/v*) FBS at 37 °C in a humidified atmosphere with 5% CO_2_. For the experimental aim, 3000 cells were plated per well onto 96-well plates and grown overnight. The cells were treated with various concentrations of pumpkin peel extracts (500–31.25 µg/mL) or an equal volume of the corresponding solvents. Following 48 h of culture, the cells were incubated for 3 h with 33 µg/mL neutral red solution in DMEM supplemented with 1% (*v*/*v*) FBS, rinsed with DPBS and lysed, using an acidified ethanol solution (50% *v*/*v* ethanol, 1% *v*/*v* acetic acid). The absorbance of the released neutral red was measured at λ = 540 nm using a FilterMax F5 microplate reader (Molecular Devices, San Jose, CA, USA). The mean measured value for the lysate from the control cells was set as 100% cellular viability and used to calculate the percentage of the viable cells following the extracts treatment.

### 3.8. Determination of the In Vitro Sun Protection Factor (SPF)

For the determination of the in vitro sun protection factor (SPF) the Mansur equation [48] was applied:

(1) $$\mathrm{SPF}=\mathrm{CF}\;\times \;\sum_{290}^{320}\mathrm{EE}\times \left(\lambda \right)\times I\times \mathrm{Abs}\left(\lambda \right)$$

where: EE (λ)—erythemal effect spectrum; I (λ)—solar intensity spectrum; Abs (λ)—absorbance of the sample; CF—correction factor (=10).

The calculation was performed using the absorbance values (λ = 290–320 nm) measured, using a DR600 UV-Vis spectrophotometer (Hach Lange, Wrocław, Poland) and the EE × I values determined, by Sayre [55].

### 3.9. Statistical Analysis

The results were considered statistically significant when *p* < 0.05. The data expressed on a quantitative scale, were presented as mean with a standard deviation (SD). Depending on the result of the Shapiro–Wilk test (assessment of the compliance with the normal distribution), the Pearson’s or Spearman correlation analysis were used. The statistical significance between the results obtained for the different extracts were analyzed using a one-way ANOVA followed by Tukey’s test. The statistical analyses were performed using Statistica software (v13.3, StatSoft, Kraków, Poland). Based on the results from the HPLC-ESI-QTOF-MS/MS platform, the chemometric analyzed were performed directly on the injection files transformed to cef format, using the Agilent Mass Profiler Professional Software (version 15.1-build 15.1.20045.0 by Agilent Technologies Inc., Santa Clara, CA, USA). The principal component analysis and cluster analysis were performed on the basis of the 781 *m/z* signals detected.

## 4. Conclusions

The studies showed that peels from various pumpkin (*Cucurbita* spp.) cultivars, considered by the food industry as by-products, might be used as a valuable source of active compounds with cosmetic properties. By using an eco-friendly extraction method and “green solvents” (water, 20% (*v*/*v*) propylene glycol in a water mixture or 70% (*v*/*v*) ethanol) obtained pumpkin peel extracts that might be directly used in cosmetic formulations. In vitro studies using human keratinocytes showed that all extracts were not cytotoxic up to the concentration of 1000 µg/mL and thus might be considered as non-irritant for the skin cells. The performed studies and statistical analysis showed that the type of solvent used for the extraction of the pumpkin peels has a significant impact on its phytochemical content and cosmetic-related activities. Among the analyzed extracts, the most interesting were the W extracts, as they contained the highest amounts of flavonoids, showed the highest antioxidant potential in the ABTS and DPPH scavenging assays, and the most significant in vitro SPF values.

On the basis of the presented data, it is difficult to indicate the pumpkin cultivar whose peel extract shows the greatest cosmetic potential as the extracts from cultivars of the same species (e.g., *C. moschata* ‘Nelson’ and ‘Muscat’) showed both the highest and lowest values for the analyzed parameters. It is however worth noting that the significant variations in the phytochemical content and biological activities, described in the scientific literature for the extracts from the flesh of pumpkin cultivars are also detectable with respect to the peel extracts. The authors believe that the presented study will contribute to the more sustainable utilization of natural resources.

## Figures and Tables

**Table 1:** The content of total phenolics, flavonoids, and protein in *Cucurbita* spp. peel extracts.

Species/Variety	Extract	Total Phenolics (mg GAE/g dw)	Flavonoids (mg QE/g dw)	Protein (mg BSA/g dw)
*C. maxima*/Halloween	W	12.116 ± 0.233 ^a^	5.032 ± 0.910 ^a^	29.892 ± 3.513 ^a^
	HG	13.920 ± 0.153 ^b^	3.967 ± 0.269 ^ab^	25.841 ± 4.719 ^a^
	E	17.599 ± 0.124 ^c^	3.114 ± 0.558 ^b^	7.871 ± 0.535 ^b^
*C. maxima*/Hokkaido	W	17.336 ± 0.249 ^a^	7.108 ± 0.120 ^a^	47.470 ± 5.277 ^a^
	HG	12.593 ± 0.493 ^b^	2.967 ± 0.081 ^b^	22.128 ± 4.421 ^b^
	E	4.623 ± 0.082 ^c^	3.629 ± 0.258 ^c^	15.588 ± 0.925 ^b^
*C. moschata*/Butternut	W	11.005 ± 0.179 ^a^	5.032 ± 0.123 ^a^	18.618 ± 0.385 ^a^
	HG	9.879 ± 0.737 ^b^	4.542 ± 0.099 ^b^	10.722 ± 0.578 ^b^
	E	6.575 ± 0.109 ^c^	3.381 ± 0.055 ^c^	5.136 ± 0.545 ^c^
*C. moschata*/Nelson	W	16.871 ± 0.382 ^a^	4.294 ± 0.254 ^a^	68.695 ± 7.790 ^a^
	HG	10.646 ± 0.386 ^b^	3.036 ± 0.123 ^b^	16.692 ± 4.022 ^b^
	E	16.599 ± 0.247 ^a^	1.738 ± 0.079 ^c^	44.427 ± 7.319 ^c^
*C. moschata*/Muscat	W	14.700 ± 0.935 ^a^	2.598 ± 0.127 ^a^	76.143 ± 4.261 ^a^
	HG	11.619 ± 0.406 ^b^	2.511 ± 0.222 ^a^	21.957 ± 3.207 ^b^
	E	7.171 ± 0.189 ^c^	1.983 ± 0.111 ^b^	25.681 ± 3.579 ^b^

W—aqueous extracts, HG—hydroglycolic extracts, E—ethanolic extracts; mean ± SD; means that do not share the same letter are significantly different with *p* < 0.05 within one species/variety.

**Table 2:** The tentatively identified compounds in *C. maxima* ‘Halloween’ peel extracts.

Ionization Mode	RT [min]	Molecular Formula	*m/z* Experimental	*m/z* Calculated	Delta [ppm]	DBE	Tentative Compound	Ref.	W	HG	E
[M-H]^−^	2.00	C_6_H_12_O_6_	179.0570	179.0561	−4.93	1	Hexahydroxycyclohexane		+	+	+
[M-H]^+^	2.05	C_5_H_11_NO_2_	118.0863	118.0863	−0.38	1	L-valine	[22]	+++	++	nd
[M-H]^−^	2.06	C_16_H_18_O_9_	353.0858	353.0878	5.66	8	Chlorogenic acid	[11]	nd	+	nd
[M-H]^+^	2.96	C_10_H_13_N_5_O_4_	268.1043	268.1040	−1.01	7	Adenosine	[23]	nd	++	+++
[M-H]^+^	4.61	C_9_H_11_NO_2_	166.0862	166.0863	0.33	5	L-phenylalanine	[22]	+++	++	+++
[M-H]^+^	12.22	C_9_H_11_NO_3_	182.0808	182.0812	2.04	5	L-tyrosine	[22]	++	nd	nd
[M-H]^+^	12.78	C_11_H_12_N_2_O_2_	205.0986	205.0972	−7.08	7	L-tryptophan	[22]	++	nd	+++
[M-H]^−^	13.87	C_7_H_6_O_3_	137.0245	137.0244	−0.60	5	p-hydroxybenzoic acid	[24]	+	++	+
[M-H]^−^	18.21	C_9_H_8_O_3_	163.0408	163.0350	−4.46	6	p-coumaric acid	[24]	+	+	+
[M-H]^−^	21.90	C_15_H_10_O_5_	269.0485	269.0455	−10.93	11	Apigenin	[25]	nd	nd	+
[M-H]^−^	36.45	C_16_H_30_O_2_	253.2207	253.2173	−13.36	2	Palmitoleic acid	[22]	+	nd	nd
[M-H]^−^	37.20	C_16_H_32_O_2_	255.2340	255.2330	−4.08	1	Palmitic acid	[22]	++	+	+
[M-H]^−^	37.66	C_18_H_32_O_2_	279.2333	279.2330	−1.24	3	Linoleic acid	[22]	++	nd	++
[M-H]^−^	37.88	C_18_H_34_O_2_	281.2495	281.2486	−3.17	2	Oleic acid	[22]	++	+	nd

Rt = retention time. Delta = difference between the experimental and calculated mass (mmu). DBE = double bond equivalents. Ref—references. W—aqueous extracts, HG—hydroglycolic extracts, E—ethanolic extracts; + 0–1,000,000; ++ 1,000,000–15,000,000; +++ >15,000,000; nd—not detected.

**Table 3:** The tentatively identified compounds in *C. maxima* ‘Hokkaido’ peel extracts.

Ionization Mode	RT [min]	Molecular Formula	*m/z* Experimental	*m/z* Calculated	Delta [ppm]	DBE	Tentative Compound	W	HG	E
[M-H]^+^	1.90	C_6_H_14_N_4_O_2_	175.1192	175.1190	−0.27	2	Arginin	nd	nd	++
[M-H]^+^	2.09	C_5_H_11_NO_2_	118.0869	118.0863	−5.51	1	L-valine	nd	++	+
[M-H]^+^	2.22	C_5_H_9_NO_2_	116.0706	116.0706	−9.52	2	Proline	nd	nd	++
[M-H]^+^	2.88	C_10_H_13_N_5_O_4_	268.1048	268.1040	−2.88	7	Adenosine	nd	+++	++
[M-H]^+^	3.97	C_9_H_11_NO_3_	182.0811	182.0812	0.38	5	L-tyrosine	nd	++	++
[M-H]^+^	4.60	C_9_H_11_NO_2_	166.0869	166.0863	−3.91	5	L-phenylalanine	nd	++	+++
[M-H]^+^	5.29	C_3_H_7_NO_2_S	122.0269	122.0270	1.04	1	Cysteine	++	nd	nd
[M-H]^−^	10.44	C_7_H_6_O_3_	137.0221	137.0244	16.79	5	p-hydroxybenzoic acid	+	+	+
[M-H]^+^	12.62	C_11_H_12_N_2_O_2_	205.0973	205.0972	−0.71	7	L-tryptophan	nd	nd	+++
[M-H]^+^	12.83	C_20_H_18_NO_4_	337.1305	337.1309	1.07	13	Berberine	nd	nd	++
[M-H]^−^	16.47	C_9_H_8_O_3_	163.0407	163.0401	−3.85	6	p-coumaric acid	+	+	+
[M-H]^−^	21.98	C_15_H_10_O_5_	269.0455	269.0455	0.17	11	Apigenin	nd	nd	+
[M-H]^−^	22.78	C_15_H_10_O_6_	285.0416	285.0405	−3.98	11	Kaempferol	nd	nd	+
[M-H]^−^	22.78	C_15_H_10_O_6_	285.0416	285.0405	−3.98	11	Luteolin	nd	nd	+
[M-H]^−^	25.34	C_18_H_36_O_2_	283.2639	283.2643	1.25	1	Stearic acid	+	+	nd
[M-H]^−^	25.39	C_18_H_34_O_2_	281.2516	281.2486	−10.61	2	Oleic acid	nd	+	+
[M-H]^−^	25.39	C_16_H_32_O_2_	255.2353	255.2330	−9.16	1	Palmitic acid	nd	+	+
[M-H]^−^	34.59	C_16_H_30_O_2_	253.2185	253.2173	−4.71	2	Palmitoleic acid	+	nd	nd
[M-H]^−^	37.69	C_18_H_32_O_2_	279.2339	279.2330	−3.38	3	Linoleic acid	+	nd	++

Rt = retention time. Delta = difference between the experimental and calculated mass (mmu). DBE = double bond equivalents. W—aqueous extracts, HG—hydroglycolic extracts, E—ethanolic extracts; + 0–1,000,000; ++ 1,000,000–15,000,000; +++ >15,000,000; nd—not detected.

**Table 4:** The tentatively identified compounds in *C. moschata* ‘Butternut’ peel extracts.

Ionization Mode	RT [min]	Molecular Formula	*m/z* Experimental	*m/z* Calculated	Delta [ppm]	DBE	Tentative Compound	W	HG	E
[M-H]^+^	1.69	C_6_H_14_N_4_O_2_	175.1201	175.1190	−6.59	2	Arginin	+	++	++
[M-H]^+^	2.03	C_5_H_9_NO_2_	116.0708	116.0706	−1.69	2	Proline	nd	+	++
[M-H]^−^	2.03	C_6_H_12_O_6_	179.0570	179.0561	−4.93	1	Hexahydroxycyclohexane	+	+	+
[M-H]^+^	2.05	C_5_H_11_NO_2_	118.0866	118.0863	−2.95	1	L-valine	+++	++	++
[M-H]^+^	2.12	C_5_H_10_N_2_O_2_	131.0801	131.0815	10.72	2	Cucurbitine	nd	nd	++
[M-H]^+^	2.87	C_10_H_13_N_5_O_4_	268.1051	268.1040	−4.00	7	Adenosine	+++	+++	+++
[M-H]^+^	2.92	C_9_H_11_NO_3_	182.0836	182.0812	−13.42	5	L-tyrosine	+	++	++
[M-H]^+^	3.93	C_3_H_7_NO_2_S	122.0257	122.0270	−0.14	1	Cysteine	++	nd	nd
[M-H]^+^	4.50	C_9_H_11_NO_2_	166.0857	166.0863	3.36	5	L-phenylalanine	+++	++	+++
[M-H]^+^	5.95	C_6_H_13_NO_5_	180.0843	180.0866	13.12	1	D-Glucopyranosylamine	nd	++	nd
[M-H]^+^	6.26	C_6_H_13_N_3_O_3_	176.1037	176.1030	−4.18	2	Citrulline	nd	+	++
[M-H]^−^	13.05	C_9_H_8_O_3_	163.0419	163.0350	−11.17	6	p-coumaric acid	+	nd	+
[M-H]^+^	13.08	C_11_H_12_N_2_O_2_	205.0962	205.0972	4.67	7	L-tryptophan	++	++	++
[M-H]^−^	13.82	C_11_H_12_O_5_	223.0641	223.0612	−12.96	6	Sinapinic acid	nd	+	nd
[M-H]^−^	13.90	C_7_H_6_O_3_	137.0255	137.0244	−7.84	5	p-hydroxybenzoic acid	+	+	+
[M-H]^−^	21.87	C_15_H_10_O_5_	269.0482	269.0455	−9.82	11	Apigenin	nd	nd	+
[M-H]^−^	22.60	C_15_H_10_O_6_	285.0435	285.0405	−10.62	11	Kaempferol	nd	nd	+
[M-H]^−^	22.60	C_15_H_10_O_6_	285.0435	285.0405	−10.62	11	Luteolin	nd	nd	+
[M-H]^−^	36.47	C_16_H_30_O_2_	253.2152	253.2173	8.28	2	Palmitoleic acid	+	+	nd
[M-H]^−^	37.21	C_16_H_32_O_2_	255.2339	255.2330	−3.69	1	Palmitic acid	+	nd	++
[M-H]^−^	37.66	C_18_H_32_O_2_	279.2352	279.2330	−8.73	3	Linoleic acid	+	+	+
[M-H]^−^	38.07	C_18_H_34_O_2_	281.2503	281.2486	−6.01	2	Oleic acid	++	nd	nd

Rt = retention time. Delta = difference between the experimental and calculated mass (mmu). DBE = double bond equivalents. W - aqueous extracts, HG—hydroglycolic extracts, E—ethanolic extracts; + 0–1,000,000; ++ 1,000,000–15,000,000; +++ >15,000,000; nd—not detected.

**Table 5:** The tentatively identified compounds in *C. moschata* ‘Nelson’ peel extracts.

Ionization Mode	RT [min]	Molecular Formula	*m/z* Experimental	*m/z* Calculated	Delta [ppm]	DBE	Tentative Compound	W	HG	E
[M-H]^−^	2.07	C_6_H_12_O_6_	179.0565	179.0561	−2.16	1	Hexahydroxycyclohexane	+	+	+
[M-H]^+^	2.12	C_5_H_11_NO_2_	118.0868	118.0863	−4.65	1	L-valine	++	++	++
[M-H]^+^	2.16	C_5_H_10_N_2_O_2_	131.0818	131.0815	−2.28	2	Cucurbitine	++	nd	++
[M-H]^+^	2.20	C_6_H_13_N_3_O_3_	176.1027	176.1030	1.53	2	Citrulline	nd	nd	nd
[M-H]^+^	2.88	C_10_H_13_N_5_O_4_	268.1046	268.1040	−2.13	7	Adenosine	nd	+++	++
[M-H]^+^	2.92	C_3_H_7_NO_2_S	122.0274	122.0270	−3.09	1	Cysteine	+	nd	nd
[M-H]^+^	3.42	C_6_H_13_NO_5_	180.0880	180.0866	−7.54	1	D-Glucopyranosylamine	+	+	nd
[M-H]^+^	4.82	C_9_H_11_NO_2_	166.0868	166.0863	−3.30	5	L-phenylalanine	++	++	++
[M-H]^+^	8.15	C_9_H_11_NO_3_	182.0837	182.0812	−13.97	5	L-tyrosine	+	++	++
[M-H]^−^	13.17	C_7_H_6_O_3_	137.0253	137.0244	−6.39	5	p-hydroxybenzoic acid	+	+	+
[M-H]^+^	14.09	C_20_H_18_NO_4_	337.1281	337.1309	8.21	13	Berberine	nd	nd	++
[M-H]^−^	18.08	C_9_H_8_O_3_	163.0425	163.0350	−14.83	6	p-coumaric acid	+	+	+
[M-H]^−^	22.47	C_15_H_10_O_5_	269.0483	269.0455	−10.19	11	Apigenin	nd	nd	+
[M-H]^−^	31.10	C_18_H_32_O_2_	279.2360	279.2330	−10.87	3	Linoleic acid	nd	+	+
[M-H]^−^	34.84	C_16_H_30_O_2_	253.2165	253.2173	3.16	2	Palmitoleic acid	+	nd	+
[M-H]^−^	37.24	C_16_H_32_O_2_	255.2345	255.2330	−6.03	1	Palmitic acid	+	nd	++

Rt = retention time. Delta = difference between the experimental and calculated mass (mmu). DBE = double bond equivalents. W - aqueous extracts, HG—hydroglycolic extracts, E—ethanolic extracts; + 0–1,000,000; ++ 1,000,000–15,000,000; +++ >15,000,000; nd—not detected.

**Table 6:** The tentatively identified compounds in C. *moschata* ‘Muscat’ peel extracts.

Ionization Mode	RT [min]	Molecular Formula	*m/z* Experimental	*m/z* Calculated	Delta [ppm]	DBE	Tentative Compound	W	HG	E
[M-H]^−^	2.03	C_6_H_12_O_6_	179.0545	179.0561	8.95	1	hexahydroxycyclohexane	+	nd	+
[M-H]^+^	2.06	C_5_H_11_NO_2_	118.0868	118.0863	−4.65	1	L-valine	++	nd	++
[M-H]^−^	2.10	C_16_H_18_O_9_	353.0882	353.0878	−1.11	8	Chlorogenic acid	+	nd	nd
[M-H]^+^	2.12	C_5_H_9_NO_2_	116.0705	116.0706	0.91	2	Proline	nd	+	++
[M-H]^+^	2.14	C_5_H_10_N_2_O_2_	131.0806	131.0815	6.95	2	Cucurbitine	++	nd	nd
[M-H]^+^	2.20	C_6_H_13_N_3_O_3_	176.1027	176.1030	1.53	2	Citrulline	nd	++	nd
[M-H]^+^	2.92	C_9_H_11_NO_3_	182.0809	182.0812	1.49	5	L-tyrosine	++	++	++
[M-H]^+^	2.88	C_10_H_13_N_5_O_4_	268.1046	268.1040	−2.13	7	Adenosine	+++	+++	+++
[M-H]^+^	4.60	C_9_H_11_NO_2_	166.0865	166.0863	−1.48	5	L-phenylalanine	+++	++	+++
[M-H]^+^	12.72	C_11_H_12_N_2_O_2_	205.0970	205.0972	0.76	7	L-tryptophan	++	nd	+++
[M-H]^−^	13.22	C_7_H_6_O_3_	137.0250	137.0244	−4.22	5	p-hydroxybenzoic acid	+	nd	+
[M-H]^−^	13.69	C_11_H_12_O_5_	223.0637	223.0612	−11.17	6	Sinapinic acid	+	nd	+
[M-H]^−^	18.06	C_9_H_8_O_3_	163.0389	163.0350	7.12	6	p-coumaric acid	+	+	+
[M-H]^−^	22.65	C_15_H_10_O_6_	285.0414	285.0405	−3.28	11	Luteolin	nd	-	+
[M-H]^−^	22.73	C_15_H_10_O_6_	285.0404	285.0405	0.22	11	Kaempferol	nd	-	+
[M-H]^−^	31.10	C_18_H_32_O_2_	279.2360	279.2330	−10.87	3	Linoleic acid	nd	+	+
[M-H]^−^	31.10	C_18_H_36_O_2_	283.2677	283.2646	−12.12	1	Stearic acid	nd	+	-
[M-H]^−^	33.29	C_16_H_32_O_2_	255.2360	255.2330	−11.89	1	Palmitic acid	nd	+	++

Rt = retention time. Delta = difference between the experimental and calculated mass (mmu). DBE = double bond equivalents. W – aqueous extracts, HG—hydroglycolic extracts, E—ethanolic extracts; + 0–1,000,000; ++ 1,000,000–15,000,000; +++ >15,000,000; nd—not detected.

**Table 7:** DPPH and ABTS scavenging activities of *Cucurbita* spp. peel extracts.

Species/Variety	Extract	ABTS Scavenging (µg TE/g dw)	DPPH Scavenging (µg TE/g dw)
*C. maxima*/Halloween	W	3.018 ± 1.085	3.272 ± 0.052 ^a^
	HG	3.855 ± 0.055	2.047 ± 0.149 ^b^
	E	4.323 ± 0.071	2.140 ± 0.169 ^b^
*C. maxima*/Hokkaido	W	4.382 ± 0.475	2.495 ± 0.055 ^a^
	HG	3.764 ± 0.048	2.701 ± 0.082 ^a^
	E	4.460 ± 0.043	1.999 ± 0.065 ^b^
*C. moschata*/Butternut	W	4.524 ± 0.231 ^a^	3.333 ± 0.004 ^a^
	HG	3.304 ± 0.011 ^b^	0.947 ± 0.026 ^b^
	E	3.600 ± 0.153 ^b^	1.386 ± 0.111 ^c^
*C. moschata*/Nelson	W	3.695 ± 0.040 ^a^	2.207 ± 0.266 ^a^
	HG	3.529 ± 0.021 ^b^	1.444 ± 0.209 ^b^
	E	3.802 ± 0.025 ^c^	2.549 ± 0.074 ^a^
*C. moschata*/Muscat	W	3.898 ± 0.042 ^a^	2.862 ± 0.283 ^a^
	HG	2.470 ± 0.041 ^b^	2.294 ± 0.103 ^b^
	E	3.552 ± 0.195 ^a^	2.473 ± 0.079 ^ab^

W—aqueous extracts, HG—hydroglycolic extracts, E—ethanolic extracts; mean ± SD; means that do not share the same letter are significantly different with *p* < 0.05 within one species/variety.

**Table 8:** In vitro Sun Protection Factor of the *Cucurbita* sp. peel extracts at 1 mg/mL.

Species/Variety	Extract		
	W	HG	E
*C. maxima* ‘Halloween’	5.83 ± 0.11 ^a^	3.06 ± 0.13 ^b^	7.30 ± 0.35 ^c^
*C. maxima* ‘Hokkaido’	6.35 ± 0.13 ^a^	2.61 ± 0.35 ^b^	1.64 ± 0.09 ^c^
*C. moschata* ‘Butternut’	7.59 ± 0.83 ^a^	0.38 ± 0.04 ^b^	2.73 ± 0.08 ^c^
*C. moschata* ‘Nelson’	7.27 ± 0.44 ^a^	1.10 ± 0.08 ^b^	4.15 ± 0.05 ^c^
*C. moschata* ‘Muscat’	2.56 ± 0.04 ^a^	2.07 ± 0.08 ^b^	1.83 ± 0.30 ^b^
TiO_2_ (1 mg/mL)	-	-	17.34 ± 0.32

mean ± SD; means that do not share the same letter are significantly different with *p* < 0.05 within one species/variety.

**Table 9:** The results of the correlation analysis between the activity of the *Cucurbita* spp. peel extracts and the content of phenolic compounds, flavonoids and proteins.

	W Extracts	HG Extracts	E Extracts
	Flavonoids (mg QE/g dw)		
ABTS (µg TE/g dw)	0.345	0.289	0.425
DPPH (µg TE/g dw)	0.001	−0.649 **	−0.689 **
SPF	0.363	−0.341	−0.232
Cytotoxicity	−0.163	−0.320	0.121
Mushroom tyrosinase	−0.061	−0.797 ***	−0.100
Murine tyrosinase	0.553 *	0.309	−0.761 ***
	**Total polyphenols (mg GAE/g dw)**		
ABTS (µg TE/g dw)	−0.229	0.651 **	−0.039
DPPH (µg TE/g dw)	−0.808 ***	0.694 **	0.430
SPF	−0.047	0.932 ***	0.831 ***
Cytotoxicity	0.400	−0.035	−0.482
Mushroom tyrosinase	−0.147	0.038	−0.165
Murine tyrosinase	−0.236	0.790 ***	0.218
	**Proteins**		
ABTS (µg TE/g dw)	−0.426	0.49	−0.029
DPPH (µg TE/g dw)	−0.721 **	0.713 **	0.850 ***
SPF	−0.443	0.797 ***	−0.157
Cytotoxicity	0.516 *	0.094	0.036
Mushroom tyrosinase	−0.016	0.371	0.229
Murine tyrosinase	−0.550 *	0.486	0.618 *

* *p* < 0.05, ** *p* < 0.01, *** *p* < 0.001; the underlined correlation coefficients—Spearman correlations, the not underlined correlation coefficients—Pearson correlations.

## Data Availability

The data are included in the manuscript.

## Supplementary Materials

The following supporting information can be downloaded at: https://www.mdpi.com/article/10.3390/molecules27217618/s1, Table S1. The fingerprints of the analyzed extracts recorded in the negative and positive ionization modes by HPLC-ESI-QTOF-MS/MS. Table S2. The selected MS/MS fragmentation spectra of the tentatively identified metabolites.

## Sample Availability

Samples of dried *Cucurbita* spp. peels are available from the authors.
