# Supplementary material for: Assessment of Cucurbita spp. Peel Extracts as Potential Sources of Active Substances for Skin Care and Dermatology
Source: Molecules. 2022 Nov 6;27(21):7618. doi: 10.3390/molecules27217618 (PMC9657783; doi:10.3390/molecules27217618)
Supplement: Supplementary file 1 [file molecules-27-07618-s001.zip › molecules-2004957-supplementary.pdf]

molecules

MDPI

*Supplementary file for the Manuscript:*

# Assessment of *Cucurbita* spp. Peel Extracts as Potential Sources of Active Substances for Skin Care and Dermatology

**Katarzyna Gaweł-Bęben, Karolina Czech, Marcelina Strzępek-Gomółka, Marcin Czop, Monika Szczepanik, Anna Lichtarska and Wirginia Kukuła-Koch**

**Table S1.** The fingerprints of the analyzed extracts recorded in negative and positive ionization mode by HPLC-ESI-QTOF-MS/MS.

### *C. maxima* 'Hokkaido' W Extract (negative and positive)

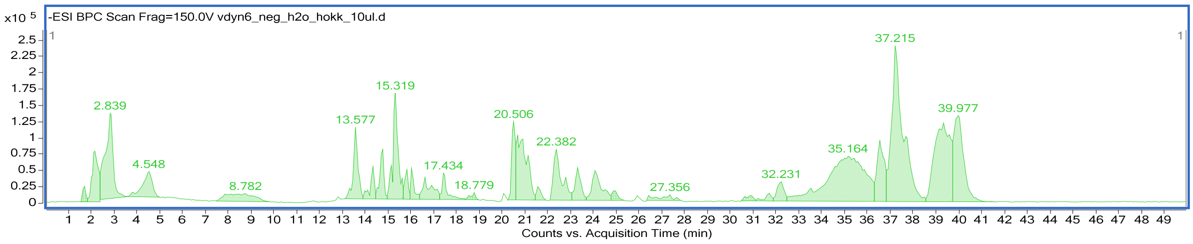


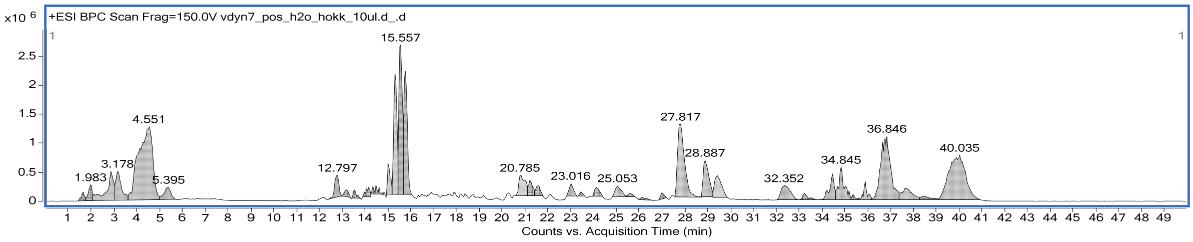


### *C. maxima* 'Hokkaido' WG Extract (negative and positive)

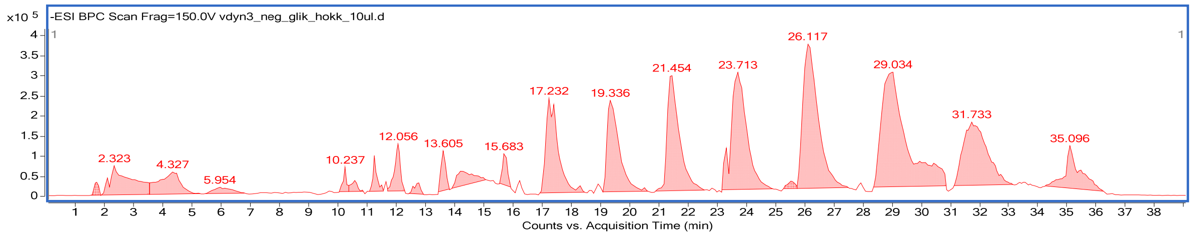


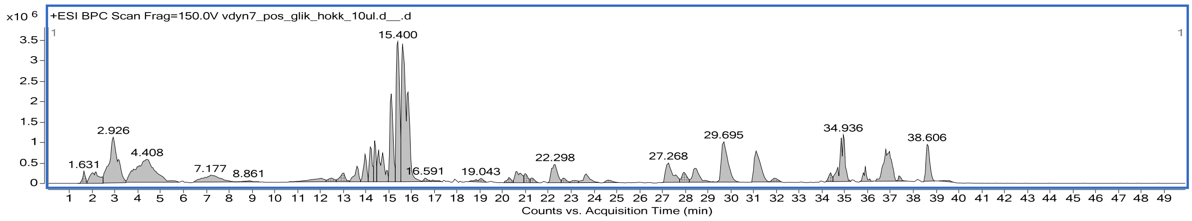


### *C. maxima* 'Hokkaido' E Extract (negative and positive)

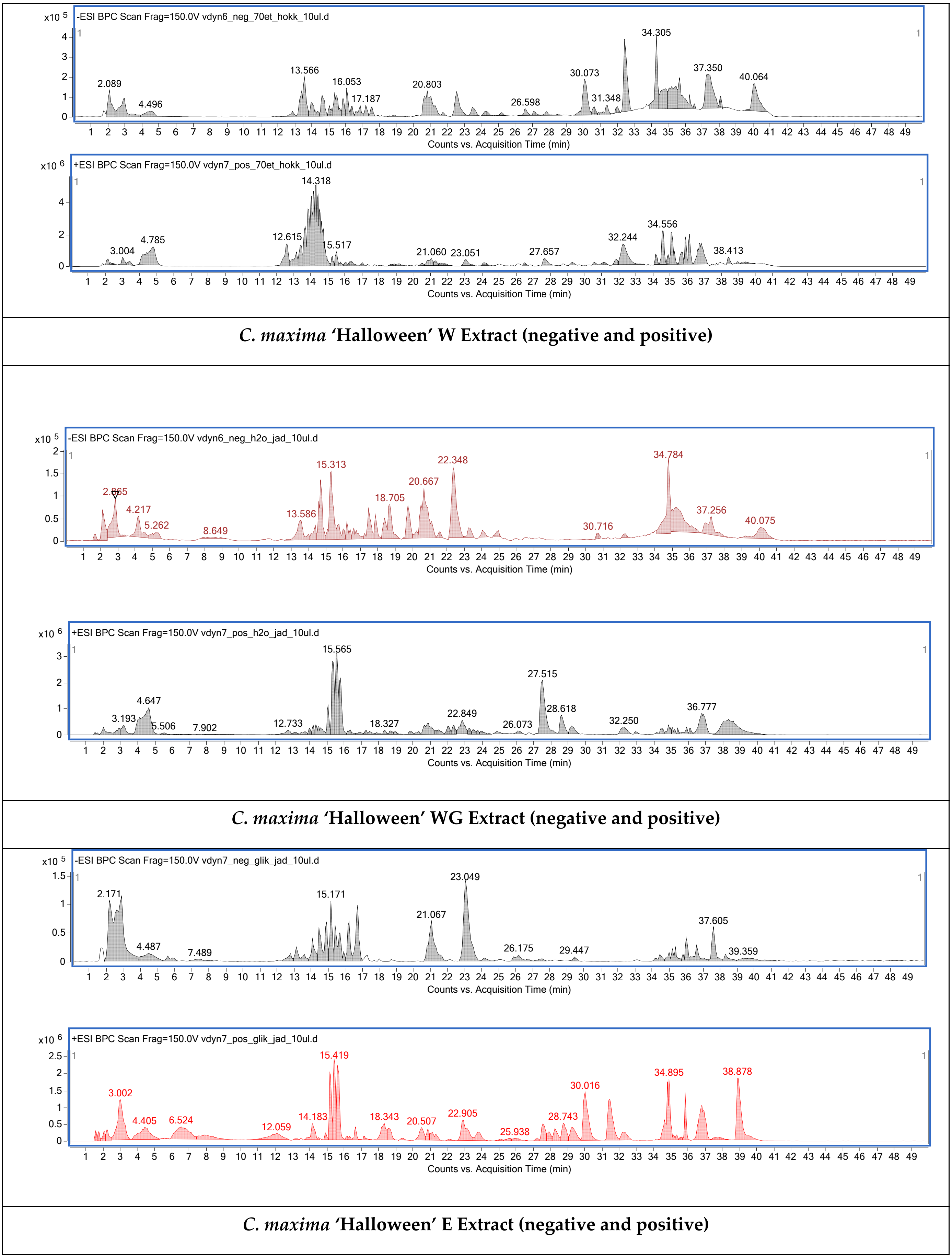
-ESI BPC Scan Frag=150.0V vdyn6_neg_70et_hokk_10ul.d
x10 5
2.089
4.496
13.566
16.053
17.187
20.803
26.598
30.073
31.348
34.305
37.350
40.064
Counts vs. Acquisition Time (min)
+ESI BPC Scan Frag=150.0V vdyn7_pos_70et_hokk_10ul.d
x10 6
3.004
4.785
12.615
14.318
15.517
21.060
23.051
27.657
32.244
34.556
38.413
Counts vs. Acquisition Time (min)
C. maxima 'Halloween' W Extract (negative and positive)
-ESI BPC Scan Frag=150.0V vdyn6_neg_h2o_jad_10ul.d
x10 5
2.865
4.217
5.262
8.649
13.586
15.313
18.705
20.667
22.348
30.716
34.784
37.256
40.075
Counts vs. Acquisition Time (min)
+ESI BPC Scan Frag=150.0V vdyn7_pos_h2o_jad_10ul.d
x10 6
3.193
4.647
5.506
7.902
12.733
15.565
18.327
22.849
26.073
27.515
28.618
32.250
36.777
Counts vs. Acquisition Time (min)
C. maxima 'Halloween' WG Extract (negative and positive)
-ESI BPC Scan Frag=150.0V vdyn7_neg_glik_jad_10ul.d
x10 5
2.171
4.487
7.489
15.171
21.067
23.049
26.175
29.447
37.605
39.359
Counts vs. Acquisition Time (min)
+ESI BPC Scan Frag=150.0V vdyn7_pos_glik_jad_10ul.d
x10 6
3.002
4.405
6.524
12.059
14.183
15.419
18.343
20.507
22.905
25.938
28.743
30.016
34.895
38.878
Counts vs. Acquisition Time (min)
C. maxima 'Halloween' E Extract (negative and positive)

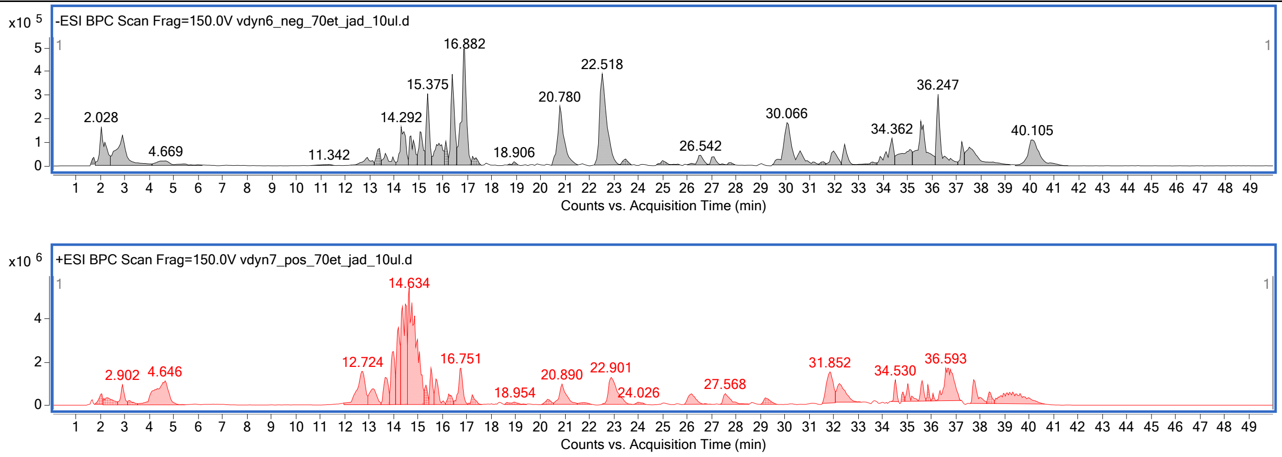


***C. moschata*** **'Muscat' W Extract (negative and positive)**

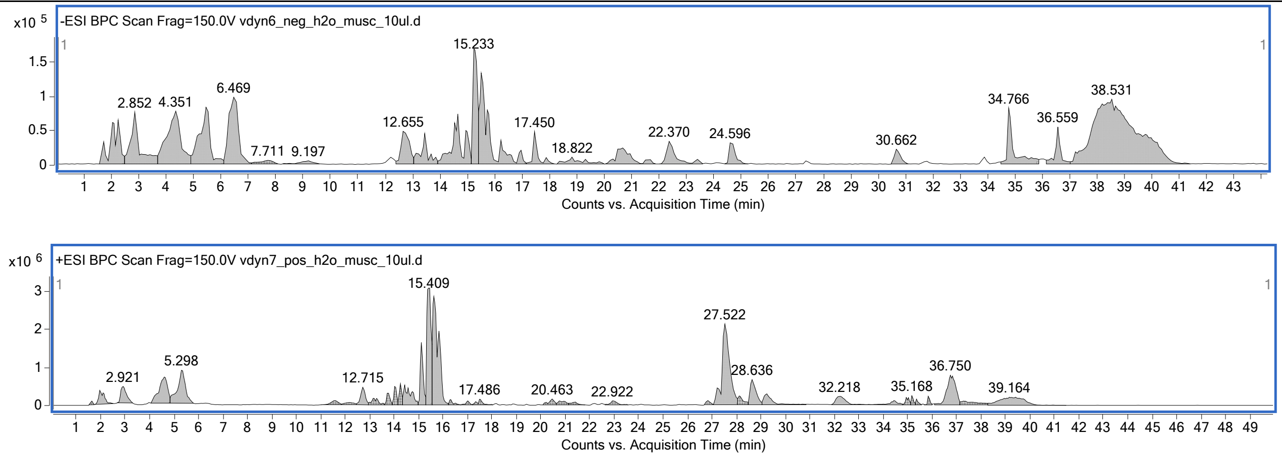


***C. moschata*** **'Muscat' WG Extract (negative and positive)**

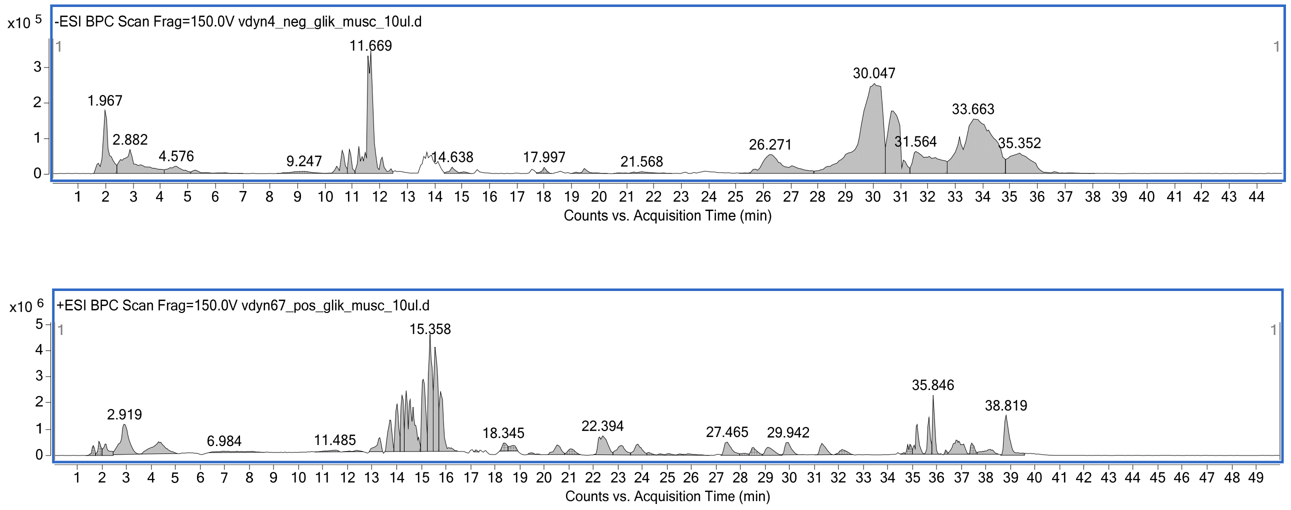


***C. moschata*** **'Muscat' E Extract (negative and positive)**

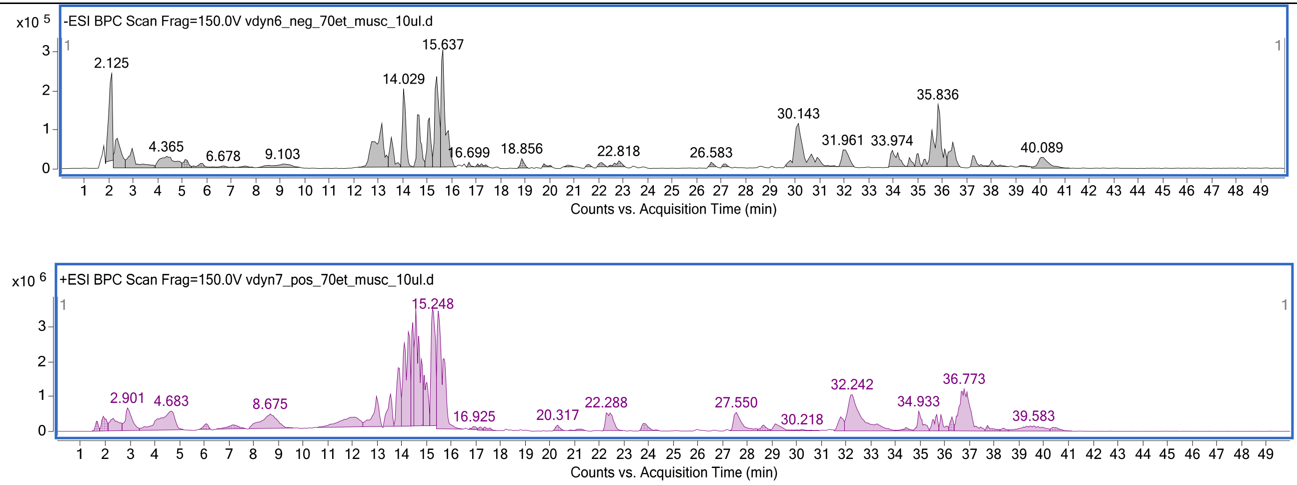


***C. moschata*** **'Nelson' W Extract (negative and positive)**

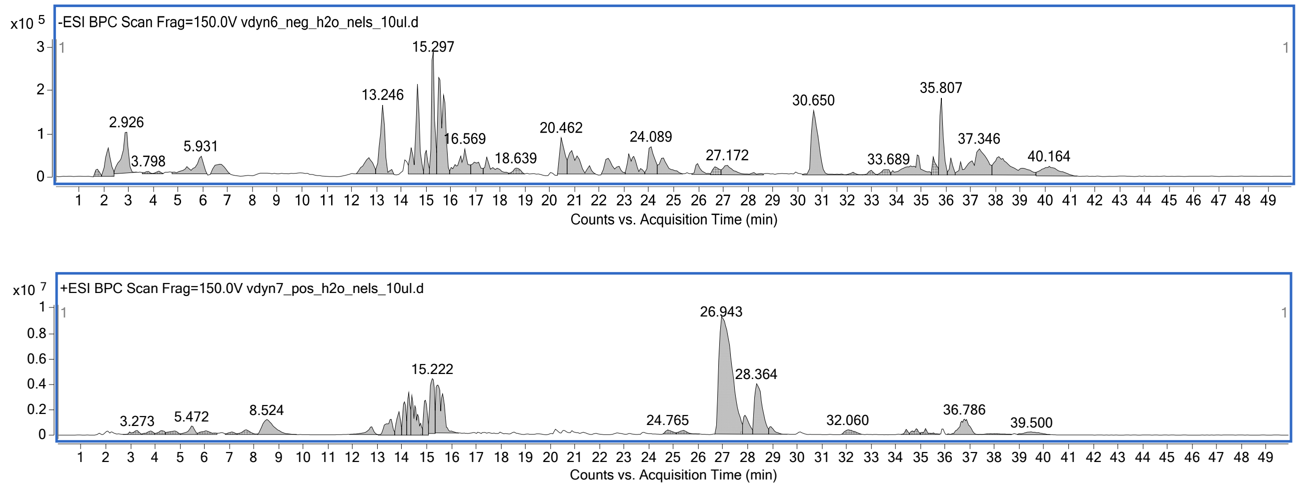


***C. moschata*** **'Nelson' WG Extract (negative and positive)**

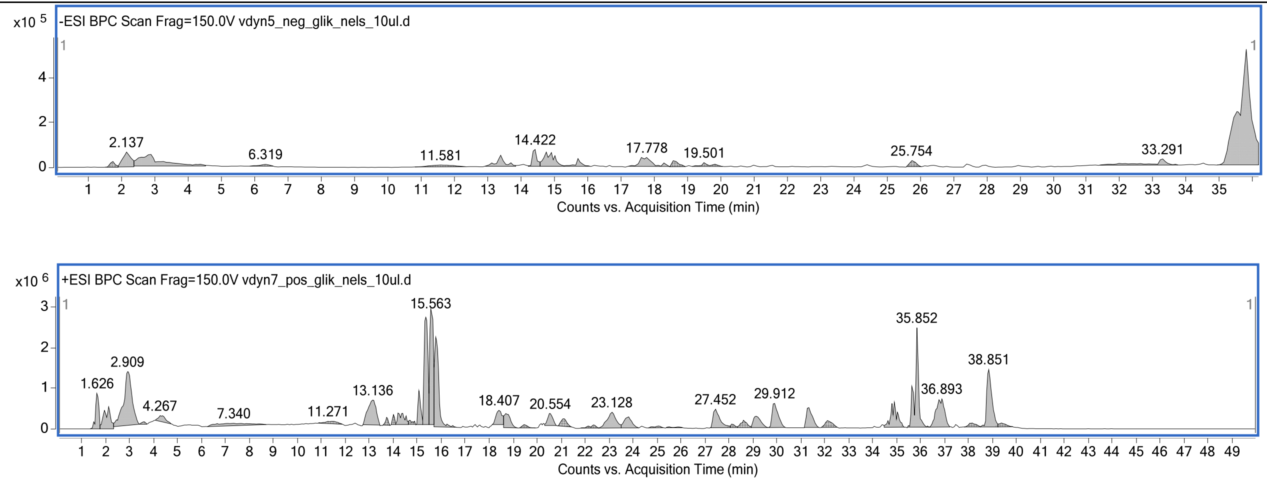


***C. moschata*** **'Nelson' E Extract (negative and positive)**

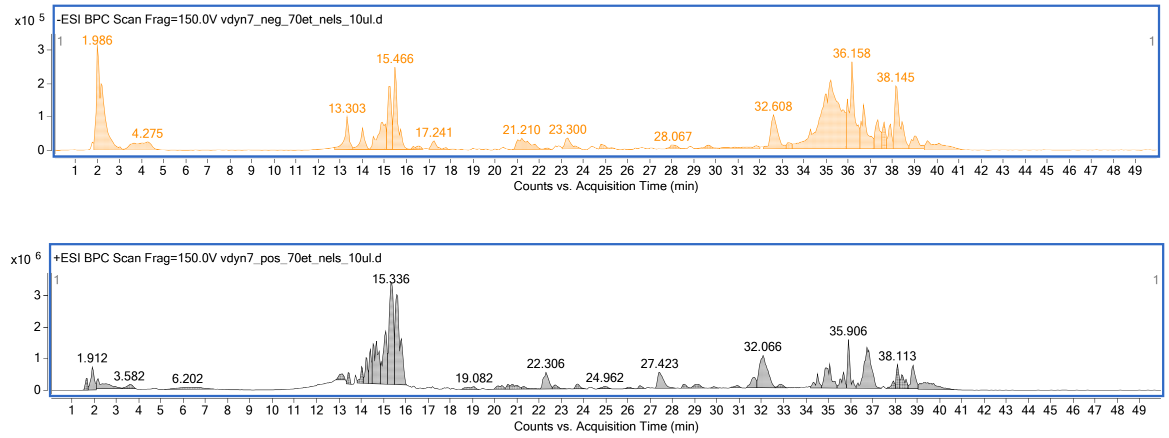


*C. moschata* **'Butternut' W Extract (negative and positive)**

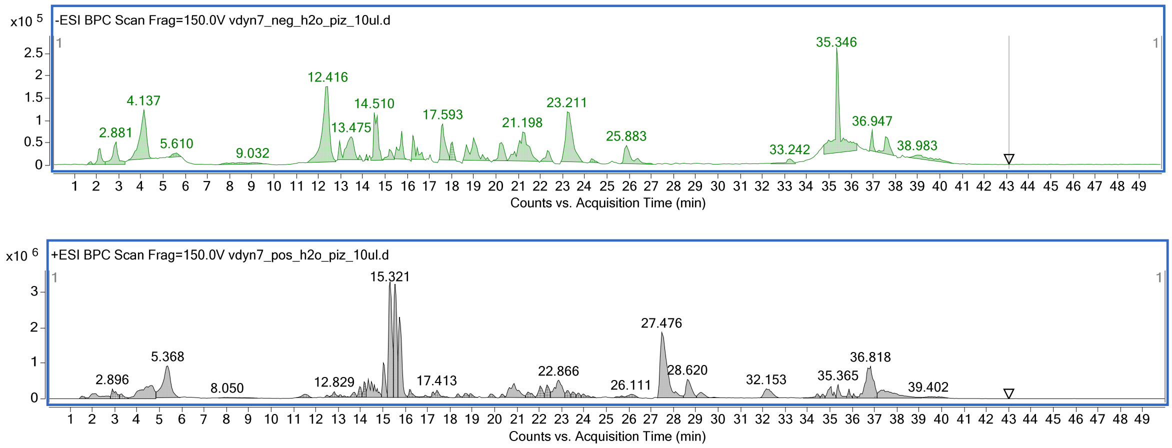


*C. moschata* **'Butternut' WG Extract (negative and positive)**

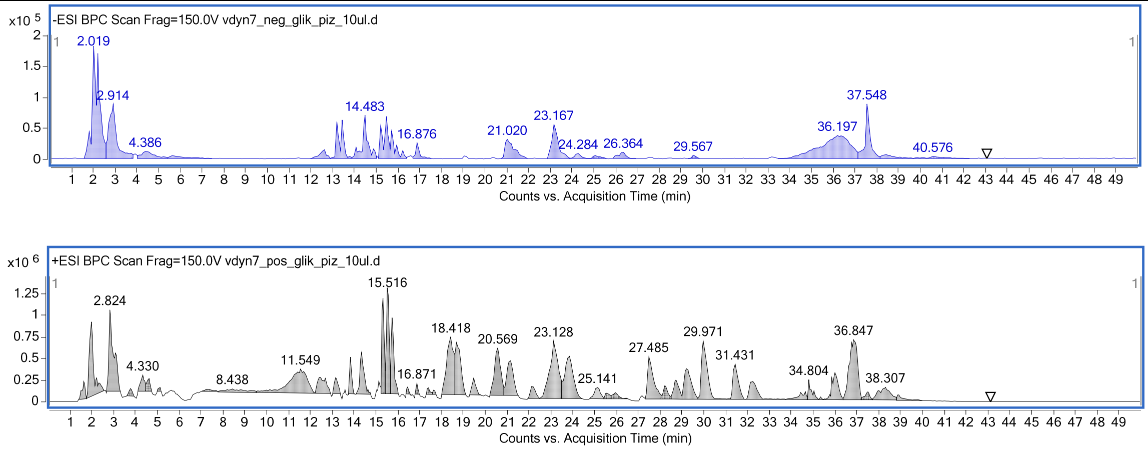


*C. moschata* **'Butternut' E Extract (negative and positive)**

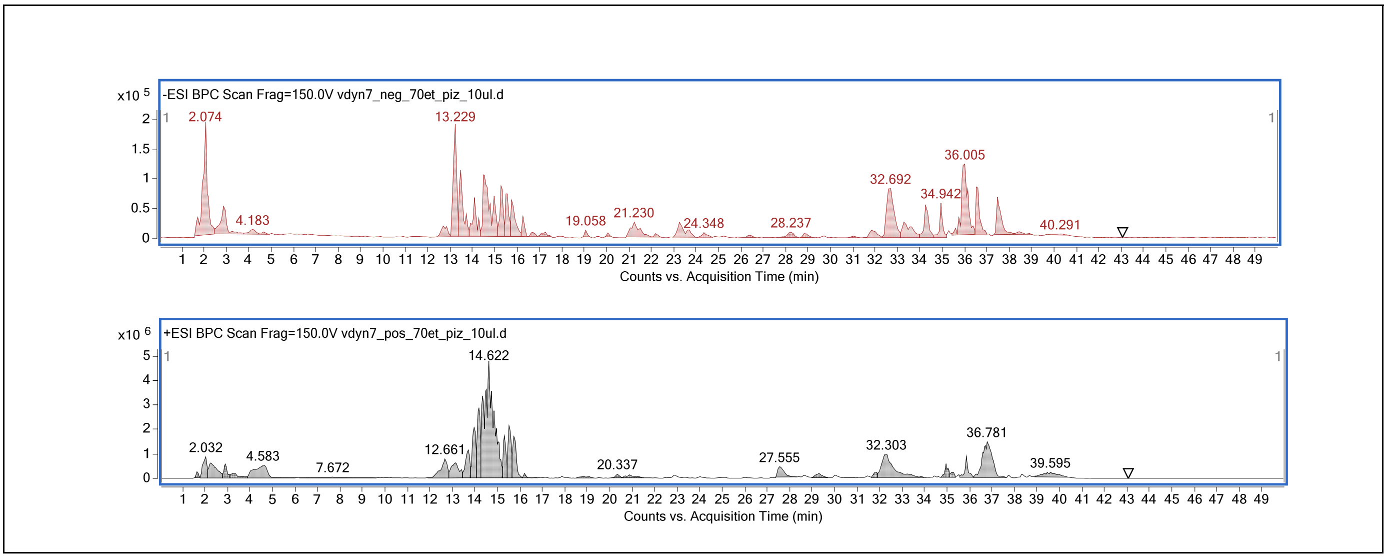
x10 5
-ESI BPC Scan Frag=150.0V vdyn7_neg_70et_piz_10ul.d
2.074
13.229
4.183
19.058
21.230
24.348
28.237
32.692
34.942
36.005
40.291
Counts vs. Acquisition Time (min)
x10 6
+ESI BPC Scan Frag=150.0V vdyn7_pos_70et_piz_10ul.d
14.622
2.032
4.583
7.672
12.661
20.337
27.555
32.303
36.781
39.595
Counts vs. Acquisition Time (min)

**Table S2.** The selected MS/MS fragmentation spectra of the tentatively identified metabolites.

<table>
<tr><th>proposed compounds</th><th>MS/MS spectra</th></tr>
<tr><td rowspan="2">L-phenylalanine</td><td>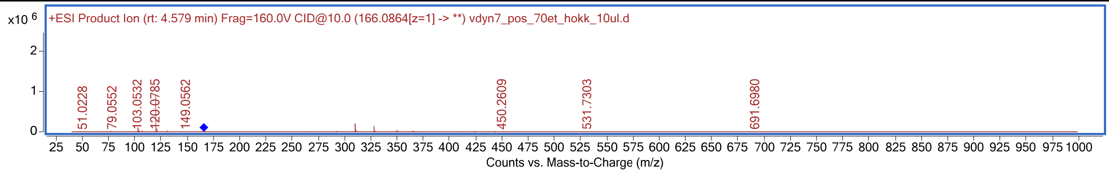
</td></tr>
<tr><td>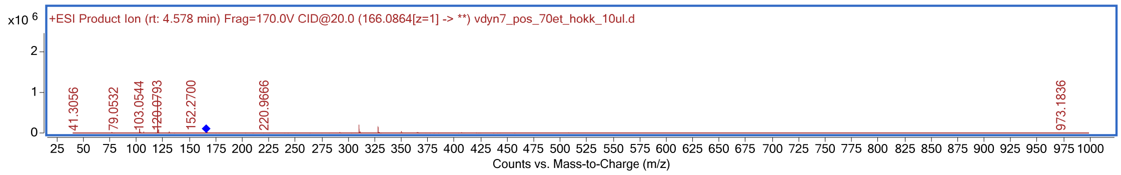
</td></tr>
<tr><td rowspan="2">Adenosine</td><td>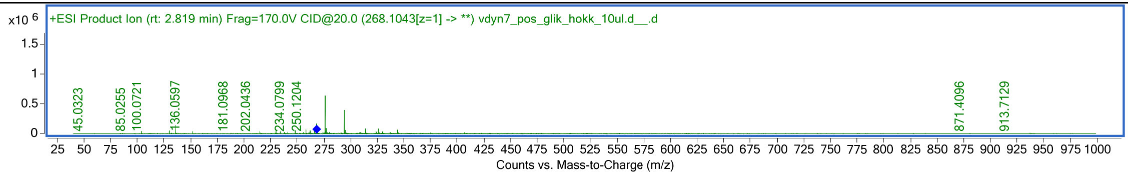
</td></tr>
<tr><td>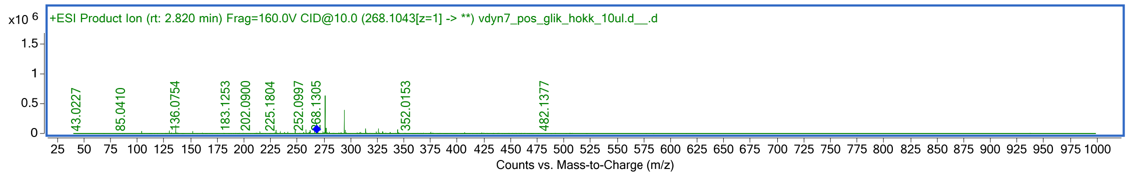
</td></tr>
<tr><td rowspan="2">L-tryptophan</td><td>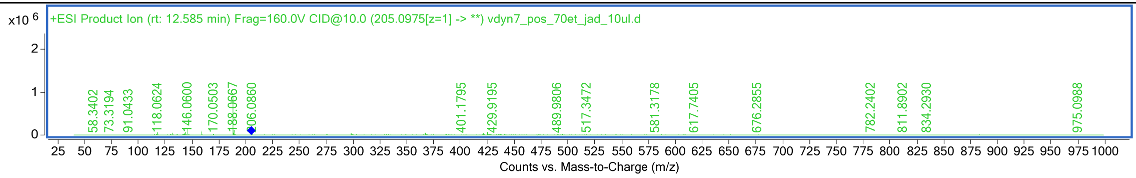
</td></tr>
<tr><td>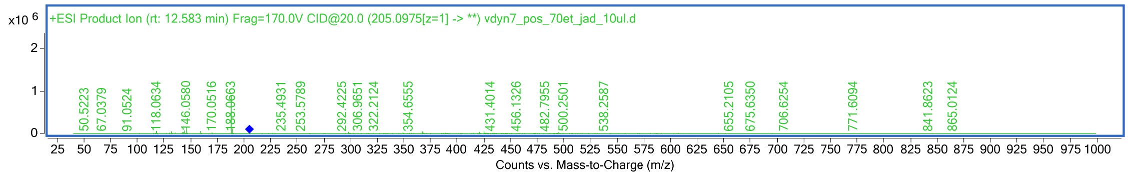
</td></tr>
<tr><td rowspan="2">L-valine</td><td>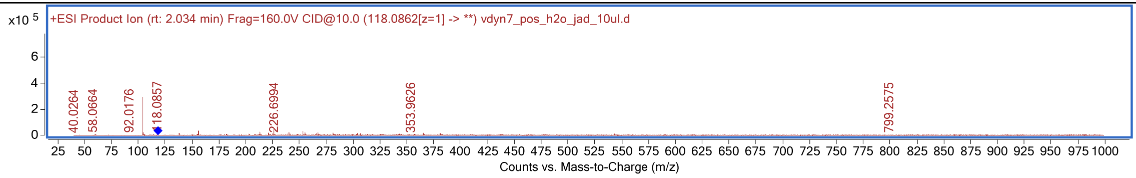
</td></tr>
<tr><td>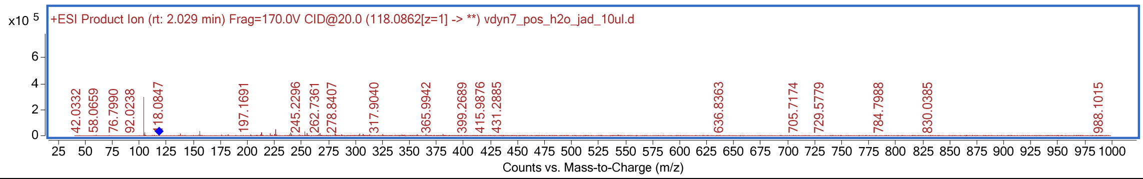
</td></tr>
</table>

| | |
|---|---|
| Adenosine | 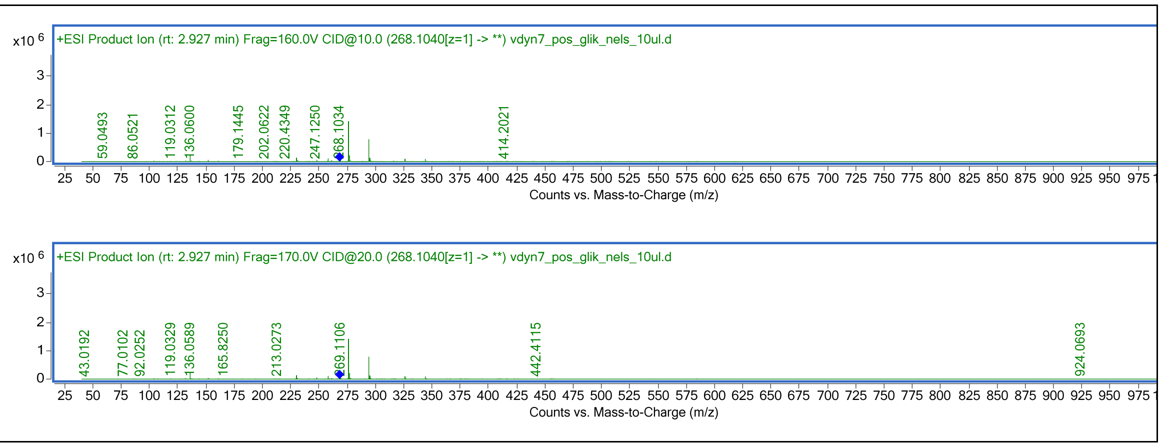 |


x10 6
+ESI Product Ion (rt: 2.927 min) Frag=160.0V CID@10.0 (268.1040[z=1] -> **) vdyn7_pos_glik_nels_10ul.d
59.0493
86.0521
119.0312
136.0600
179.1445
202.0622
220.4349
247.1250
268.1034
414.2021
Counts vs. Mass-to-Charge (m/z)
x10 6
+ESI Product Ion (rt: 2.927 min) Frag=170.0V CID@20.0 (268.1040[z=1] -> **) vdyn7_pos_glik_nels_10ul.d
43.0192
77.0102
92.0252
119.0329
136.0589
165.8250
213.0273
269.1106
442.4115
924.0693
Counts vs. Mass-to-Charge (m/z)
